# Bidirectional regulation of KEAP1 BTB domain-based sensor activity

**DOI:** 10.1016/j.redox.2025.103885

**Published:** 2025-10-08

**Authors:** Takafumi Suzuki, Kenji Takagi, Tatsuro Iso, Huaichun Wen, Anqi Zhang, Tetsuya Hatakeyama, Hiraku Oshima, Tsunehiro Mizushima, Masayuki Yamamoto

**Affiliations:** a Department of Biochemistry & Molecular Biology, Tohoku Medical Megabank Organization, Tohoku University, 2-1 Seiryo-machi, Aoba-ku, Sendai, 980-8573, Japan; b Advanced Research Center for Innovations in Next-Generation Medicine (INGEM), Tohoku University, 2-1 Seiryo-machi, Aoba-ku, Sendai, 980-8573, Japan; c National Institute of Technology, Tsuyama College, 624-1, Numa, Tsuyama-city, Okayama, 708-8509, Japan; d Department of Science, Graduate School of Science, University of Hyogo, 3-2-1 Kouto, Kamigori-cho, Ako-gun, Hyogo, 678-1297, Japan; e Department of Science, Graduate School of Science, University of Hyogo, 2167, Shosha, Himeji, Hyogo, 671-2280, Japan; f Bio-dynamics Research Center, University of Hyogo, 3-2-1 Kouto, Kamigori-cho, Ako-gun, Hyogo, 678-1297, Japan

**Keywords:** KEAP1, NRF2, BTB domain, Stress sensor, Ubiquitin ligase

## Abstract

The KEAP1-CUL3 ubiquitin ligase regulates protein stability of transcriptional factor NRF2 and plays critical roles in cellular stress response. The BTB domain of KEAP1 functions as a sensor for electrophilic chemicals. However, the precise mechanisms by which electrophiles are recognized and inhibit BTB activity remain unclear. Here, we show that electrophilic modification alters the spatial arrangement of the BTB homodimer, regulating its ligase activity. Co-crystal structural analyses and functional studies using potent NRF2-inducing CDDO-derivatives, synthetic electrophilic compounds structurally related to clinically approved molecules such as Omaveloxolone, revealed that the key sensor residue, Cys151, resides in a structurally elaborate environment within the BTB domain. Modification of Cys151 by NRF2 inducers changes the spatial configuration of the CUL3-binding sites in the BTB homodimer, reducing KEAP1-CUL3 complex affinity. In contrast, a Cys151-targeting NRF2 inhibitor induces an opposite rearrangement of the BTB homodimer. This study elucidates the molecular mechanism by which the BTB domain finely regulates KEAP1-CUL3 ubiquitin ligase activity.

## Highlights

• Modification of KEAP1-Cys151 provokes conformational changes of the BTB homodimer.
• The BTB dimer alterations change the spatial configuration of the CUL3-binding sites.
• Bidirectional alterations in the BTB structure regulate cytoprotective NRF2 activity.

## Graphical abstract

**Figure:**
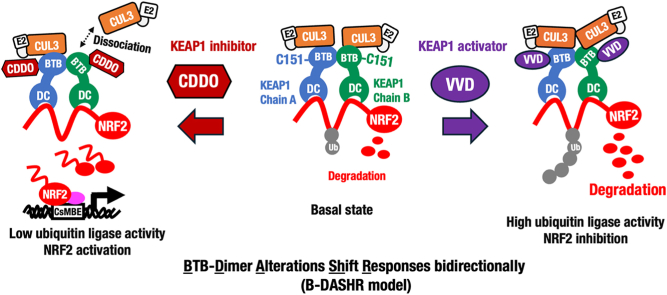

## 1 Introduction

The transcription factor NRF2 (NF-E2-related factor 2) plays a central role in the inducible cytoprotective response to oxidative insults [1,2]. Under basal unstressed conditions, NRF2 protein levels are maintained at relatively low level due to the constitutive ubiquitination of NRF2 by KEAP1 (Kelch-like ECH-associated protein 1), an adaptor component of a CUL3 (Cullin 3)-based ubiquitin E3 ligase complex. KEAP1 targets NRF2 for proteasomal degradation [[3], [4], [5]]. Upon exposure to reactive oxygen species or electrophiles, the ubiquitin-conjugation activity of KEAP1 and consequently ubiquitination of NRF2 is repressed, leading to the stabilization and nuclear translocation/accumulation of NRF2 [6]. This is followed by the upregulation of detoxifying and antioxidant gene expressions [7,8].

KEAP1 interacts with the Neh2 (NRF2-ECH homology domain 2) degron domain of NRF2 [3]. The stoichiometry of KEAP1 and NRF2 is 2:1 within the KEAP1-NRF2 complex [[9], [10], [11]]. Two site binding of NRF2 and KEAP1 homodimer is critical for the ubiquitination reaction of NRF2 by the KEAP1-CUL3 ubiquitin E3 ligase complex [9]. The overall structure of the KEAP1 homodimer resembles a cherry-bob, with two globular units (DC domains, or the double glycine repeat and the C-terminal domains) connected by homodimerizing BTB (bric-a-brac, tramtrack, and broad complex) domains, and IVRs (intervening regions) linking the DC and BTB domains [12]. While the DC domain is responsible for interaction with NRF2, the BTB contributes not only to homodimerization but also to interaction with CUL3 [4]. The BTB domain is important for the regulation of NRF2.

A multitude of NRF2 inducers have been reported, most of which are electrophilic and readily react with cysteine thiol groups within KEAP1 [13,14]. The significance of these cysteine residues has been elucidated by studies using cysteine-substituted KEAP1 mutant mice and cultured cells [6,[15], [16], [17], [18], [19]]. We have classified NRF2-inducers into at least four classes based on the functional necessity of cysteine residues in KEAP1. Class I consists of Cys151-dependent inducers; Class II, Cys288-dependent inducers; Class III, Cys151/Cys273/Cys288-selective inducers; and Class IV, Cys226/Cys613/Cys622/Cys624-preferring inducers [6,15,16]. Of these multiple classes of NRF2-inducers, Class I includes the major NRF2-inducing electrophilic chemicals, such as triterpenoid CDDO compounds [20], which contain α,β-unsaturated carbonyl groups that act as Michael acceptors, enabling them to react with KEAP1-Cys151 and activate NRF2. CDDO-Im, one of the most potent chemicals. CDDO-2P-Im and CDDO-3P-Im, two new analogs of CDDO-Im, have also been developed with better bioavailability [21].

Another CDDO (Bardoxolone) derivative, CDDO-methyl ester (CDDO-Me or Bardoxolone methyl®) was advanced to phase 3 clinical trials for diabetic nephropathy [22] (the phase 2 and 3 trials: BEAM, NCT00811889; BEACON, NCT01351675; CARDINAL, NCT03019185; PHOENIX, NCT03366337; TSUBAKI, NCT02316821; and AYAME, NCT03550443), but did not proceed further. The functional necessity of Cys151 for CDDO-Me activity has been validated [17]. In contrast, a difluoropropanamide derivative of CDDO, CDDO-DFPA (RTA-408, Omaveloxolone or SKYCLARYS®), has been approved for patients with Friedreich's ataxia [23]. Moreover, sulforaphane (SFN), dimethyl fumarate (DMF or Techfidera®), and *tert*-butylhydroquinone (tBHQ) have all been shown to act as Cys151-preferring inducers [15,19,24].

The Cys151 residue is the main sensor for electrophiles and is located within the BTB domain of KEAP1. It has been shown that modification of Cys151 inhibits the KEAP1-CUL3 interaction and prevents the ubiquitination of NRF2 [[25], [26], [27]]. However, the precise mechanisms by which modifications of the Cys151 residue in KEAP1 affect KEAP1-CUL3 activity toward NRF2 ubiquitination remain to be clarified. In this study, therefore, we investigated the co-crystal structures of the KEAP1-BTB domain complexed with potent NRF2-inducing CDDO-derivative compounds. We identified that the key sensor residue, Cys151, resides in a structurally elaborate environment within the BTB domain. A salient finding of this study is that modification of Cys151 provokes significant conformational changes in the combined structure of two chains (Chain A and Chain B) of the BTB homodimer. These structural changes alter the interaction between the BTB domain and CUL3 N-terminal structure, resulting in the reduction of the KEAP1-CUL3 ubiquitin ligase activity. Furthermore, we found that a recently introduced KEAP1 activator, which also interacts with Cys151 residue, elicited completely distinct structural changes. We report here the precise molecular basis by which KEAP1 exerts elaborate sensor function for Cys151 interacting electrophilic chemicals.

## 2 Methods

***Chemical reagents.*** 1-[2-cyano-3,12-dioxooleana-1,9(11)-dien-28-oyl] imidazole (CDDO-Im) [20] was kindly provided by Dr. Michael Sporn. CDDO was purchased from Cayman. CDDO-Me and RTA-408 were purchased from Selleck.

***Expression constructs.*** For the pull-down assay, 6xHis-TEV-tagged mouse KEAP1 full-length (1–624 a.a., WT, C151S, Y85F and C151S&Y85F) was cloned into the pET21a vector (Novagen). The 6xHis-tagged N-terminus domain (1–384 a.a., I342R/L346D) of mouse CUL3 (CUL3^NTD^) was cloned into the pCold III vector (TAKARA Bio). For the crystallization, mouse KEAP1-BTB domain (50–179 a.a., S172A) was cloned into the pET101 vector (Invitrogen). Y85F mutation was introduced into the vector.

***Crystallization and data collection****.* 6xHis-tagged KEAP1-BTB (50–179 a.a., S172A) was expressed in bacteria and purified by HisPur Ni-NTA Resin (Thermo Scientific) and HiTrap Q XL (Cytiva) as described [10]. The purified 6xHis-tagged BTB was digested with TurboTEV protease (MobiTec) and both the His-tags and the protease were removed using HisPur Ni-NTA Resin (Thermo Scientific). The untagged BTB protein was further purified by Hiload 16/600 Superdex 75 pg (Cytiva). The purity of the BTB protein was assessed by SDS-PAGE followed by staining with Oriole Fluorescent Gel Stain (Bio-Rad, #1610496).

KEAP1-BTB was concentrated to 11.0 mg/ml by ultrafiltration in a buffer containing 20 mM Tris-HCl, 100 mM NaCl, 1 mM TCEP (pH 8.0). A 1.0-μl aliquot of a concentrated protein were mixed with 1.0 μl of crystallization solution and equilibrated by hanging-drop vapor diffusion against 200 μl of reservoir solution. Crystals were obtained in a drop containing 100 mM Bis-Tris Propane (pH 6.5), 200 mM LiCl, 13 % PEG 3350, 4 % Sucrose at 4 °C.

In the process of crystallization of KEAP1 complexed with CDDO-Im or CDDO-Me, for co-crystallization with CDDO-Im, 60 μM of KEAP1 was pre-incubated overnight at 4 °C with 300 μM of CDDO-Im, then concentrated to 6.2 mg/mL using ultrafiltration. For co-crystallization with CDDO-Me, 10 μM of KEAP1 was pre-incubated overnight at 4 °C with 30 μM of CDDO-Me, then concentrated to 34.9 mg/mL using ultrafiltration. A 1.0-μl aliquot of a concentrated protein were mixed with 1.0 μl of crystallization solution and equilibrated by hanging-drop vapor diffusion against 200 μl of reservoir solution. Crystals were obtained in a drop containing 100 mM Tris-HCl (pH 8.0), 200 mM CaCl_2_, 13 % PEG3350, 2 % 2-propanol at 4 °C for CDDO-Im complex, 100 mM AAB (sodium acetate trihydrate, N-(2-acetamido)iminodiacetic acid and bicine) pH 7.0, 6 % PEG20000, 200 mM ammonium sulfate, 100 mM arginine, 100 mM glutamic acid for CDDO-Me complex, respectively. Diffraction data were collected under respective cryogenic conditions on BL44XU at SPring-8 facility in Hyogo, Japan. The data sets were processed using XDS [28].

***Structure determination and refinement***. The structure of apo KEAP1-BTB was determined by molecular replacement with MOLREP [29] from the CCP4 suite using human KEAP1-BTB (48–180 a.a.) (PDB ID: 4CXI) as a search model. Several rounds of manual fitting and refinement were carried out with the programs Coot [30], REFMAC5 [31], and PHENIX [32].

The structure of the KEAP1-BTB complexed with CDDO-Im or CDDO-Me were solved by molecular replacement performed with MOLREP [28] from the CCP4 suite or Phaser [33] with the refined apo-form KEAP1-BTB structure as a search model. Several rounds of manual fitting and refinement were carried out as well as KEAP1-BTB. The electron density maps (2F_o_-F_c_ (sigma level 1.0), F_o_-F_c_ (sigma level±3.0) and Polder (sigma level 3.0)) [34] clearly showed the presence of the CDDO-Im, but not well of the CDDO-Me. For a summary of the data reduction and refinement statistics, see Supplementary Table S1.

***AlphaFold3 analysis.*** Atomic models of full-length KEAP1 in complex with CUL3 were predicted with AlphaFold3 webserver with default settings. The amino acid sequences of mouse KEAP1 and CUL3 were used as the input. Five models were generated and the relaxed model with the highest confidence score was selected for analysis [35,36].

The structural models of the KEAP1 BTB in complex with NRF2 inducers were generated using AlphaFold3 [37]. The amino acid sequence of KEAP1 BTB S172A and the SMILES format of SFN, tBHQ, and DMF were prepared as inputs. The highest-scoring models were selected for SFN and tBHQ, while for DMF the top-ranked model in which DMF was located near Cys151 was used for analysis.

***Pull-down analysis.*** 6xHis-tagged proteins of full-length KEAP1 and CUL3^NTD^ were expressed in bacteria and purified using HisPur Ni-NTA Resin (Thermo Scientific) and HiTrap Q XL (Cytiva). 6xHis-full-length KEAP1 proteins were digested with TurboTEV protease (MobiTec) and both the His-tags and the protease were removed using HisPur Ni-NTA Resin (Thermo Scientific). The untagged full-length KEAP1 and 6xHis-tagged CUL3^NTD^ were purified by Hiload 16/600 Superdex 200 pg (Cytiva). The amounts of purified proteins were determined by using Quick Start Bradford 1xDye Reagent (Bio-Rad, #5000205) in a comparison with a BSA standard.

The purified KEAP1 protein (1 μΜ) was mixed with chemicals (1, 3 or 10 μΜ) or DMSO vehicle in washing buffer (50-mM Tris-HCl, 300-mM NaCl, 20-mM Imidazole, 10-mM GSH (pH 8.0)). After incubation for 30 min at 20 °C, the mixture was mixed with purified 6xHis-CUL3^NTD^ (1 μΜ) in a final 50-μl reaction volume. After incubation for an additional 30 min at 20 °C, the mixture was incubated with 20-μl Ni-NTA magnetic agarose beads (Qiagen) for 45 min at 20 °C, washed two times with the above washing buffer, and eluted with 25-μl elution buffer (50-mM Tris-HCl, 300-mM NaCl, 300-mM Imidazole, 10-mM GSH (pH 8.0)). Bound proteins were analyzed by SDS-PAGE, followed by staining with Oriole Fluorescent Gel Stain (Bio-Rad, #1610496). Gel image is obtained by ChemiDox XRS Plus (Bio-Rad) and analyzed by Quantity One software (Bio-Rad).

***Establishment of stable cell lines that express KEAP1.*** The PiggyBac transposon vector system (PB514B-2, System Biosciences) was used to establish stable cell lines that express HA-tagged KEAP1 cDNA. Mouse KEAP1 cDNA mutants were inserted into the PiggyBac expression vector as previously described [15,16]. Immortalized *Keap1*-null MEFs [38,39] were maintained in DMEM (1 mg/ml glucose, Wako Chemical) containing 10 % fetal bovine serum (FBS). Co-transfection of the PiggyBac expression vector plus the transposase plasmid was performed by electroporation with double 1100 V pulse for 30 ms. After 2–3 days culture, electroporated cells were selected using 2 μg/ml of puromycin for 7–10 days. Expression of RFP (red fluorescent protein) was verified under a fluorescence microscope, and several individual colonies were selected and grown for further analysis.

***RNA extraction and quantitative real-time PCR.*** Total RNAs were prepared from MEFs using a Sepazol®-RNA I Super G RNA extraction kit (Nacalai). The cDNAs were synthesized from total RNA using ReverTra Ace® qPCR RT Master Mix with gDNA Remover (TOYOBO). Real-time quantitative PCR was performed using QuantStudio (Applied Biosystems). The primer and probe sequences used for detecting *Nqo1* and *Gclc* have been described previously [40].

***Immunoblotting.*** Whole cell extracts were prepared in a sample buffer (20 % glycerol, 4 % SDS, 0.125 M Tris-HCl [pH 6.8], and 0.2 M dithiothreitol) from MEFs treated with NRF2-inducing chemicals for 3 h. The protein samples were subjected to 8 % SDS-polyacrylamide gel electrophoresis (SDS-PAGE) and electro-transferred to PVDF membranes. Specific protein signals were detected by anti-NRF2 (1:200 dilution) [41], anti-KEAP1 (2007; 1:200 dilution) [42] or anti-α-Tubulin (T9026, Sigma DM1A; 1:1000 dilution) antibodies.

***Protein structure accession number***. The atomic coordinates have been deposited in the PDB and assigned accession number as BTB-WT (9UJG), CDDO-Im-modified BTB (9UJI) and CDDO-Me-modified BTB (9UJJ).

## 3 Results

**Fig. 1:**
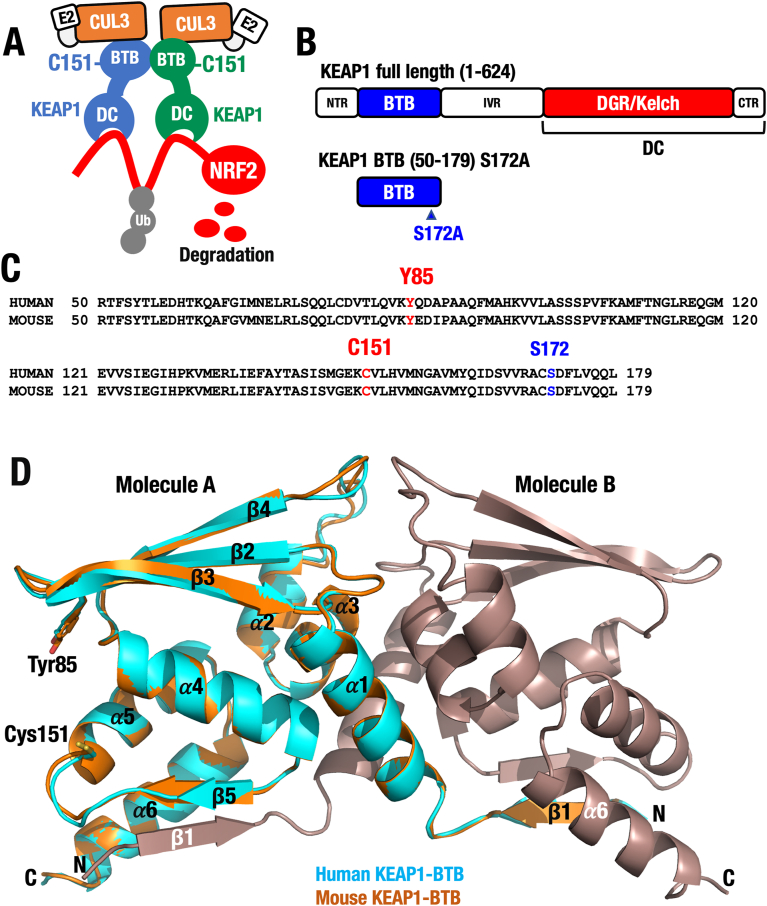
**Crystal structure of the KEAP1-BTB domain. (A)** The KEAP1-CUL3 ubiquitin E3 ligase regulates NRF2 stability. Cys151 in KEAP1-BTB domain acts as the primary electrophile sensor. **(B)** Structure of full-length KEAP1 and BTB^S172A^ domain (50–179) used for crystal structure analyses. (**C**) Alignment of human and mouse KEAP1-BTB domain. Note the conservation of Cys151, Tyr85, and Ser172. (**D**) Comparison of KEAP1-BTB structures between human (PDB: 4CXI) and mouse (PDB: 9UJG).

**Structure of the KEAP1-BTB homodimer.** We have demonstrated that the BTB domain of KEAP1 plays a critical role in the induction of NRF2 in response to a group of electrophiles [6,15,19,24]. This NRF2 induction is achieved by weakening the E3 ubiquitin ligase activity composed of KEAP1 and CUL3. The key player in the BTB domain's sensor activity for electrophiles is the reactive cysteine residue Cys151. We have demonstrated this fact by generating a Cys151-to-serine substitution mutant mouse (C151S) [15]. However, the mechanisms by which the main sensor, Cys151 in the BTB domain, recognizes electrophilic NRF2-inducing chemicals and how these electrophiles impair the E3 ubiquitin ligase activity remain to be elucidated. To address this question, it is essential to clarify the protein structure of the KEAP1-BTB domain, including its interaction with CUL3 (Fig. 1A).

Elucidation of the full KEAP1 structure or the KEAP1-CUL3 complex structure has not yet been accomplished due to various technical challenges. However, pioneering structural studies of the DC domain [43,44] and BTB domain [25] have been reported. To facilitate structure-function analysis of the BTB domain, we generated crystals of mouse KEAP1-BTB domain (50–179 amino acids), following the approach used in the crystal structural analysis of the human KEAP1-BTB domain [25], which includes an S172A substitution that enhanced crystallization (Fig. 1B).

Of note, amino acid alignment of human and mouse BTB domains revealed that these domains are highly conserved, differing at only four residues (Fig. 1C). Based on this conservation, we processed to crystalize the mouse KEAP1-BTB domain. The dimeric form of the BTB domain was purified to homogeneity (Supplementary Fig. S1), and the protein was successfully crystallized. We determined the crystal structure of the mouse KEAP1-BTB domain at 2.8 Å resolution using SPring-8 (Fig. 1D). The mouse BTB domain consists of a three-stranded β-sheet and six α-helices, forming a homodimer. The two domains related by a crystallographic two-fold axis. As expected, the structure of the mouse BTB domain closely resembles that of the human KEAP1-BTB domain (PDB ID: 4CXI) with a root-mean-square deviation (r.m.s.d.) of 0.48 Å for 130 Cα atoms, suggesting that the function of the BTB domain is conserved between human and mouse. It should be noted that an antiparallel β-sheet is formed between strands 51–55 (β1) of Molecule B (shown in brown) and 144–147 (β5) of Molecule A (orange), located near helix 163–178 (α6) of Molecule A. A similar structure is observed in the reciprocal region of the homodimer.

**Co-crystal structure of the KEAP1-BTB domain with CDDO-Im**. The BTB domain of KEAP1 plays a key role in the induction of NRF2 by a group of electrophilic inducers. Following the successful crystallization of the BTB domain, we next attempted co-crystallization with an electrophilic inducer. For this purpose, we selected CDDO-Im, one of the most potent NRF2-inducing compounds in the original CDDO family [20], which has also been widely used in disease model mouse studies [[45], [46], [47], [48], [49]].

**Fig. 2:**
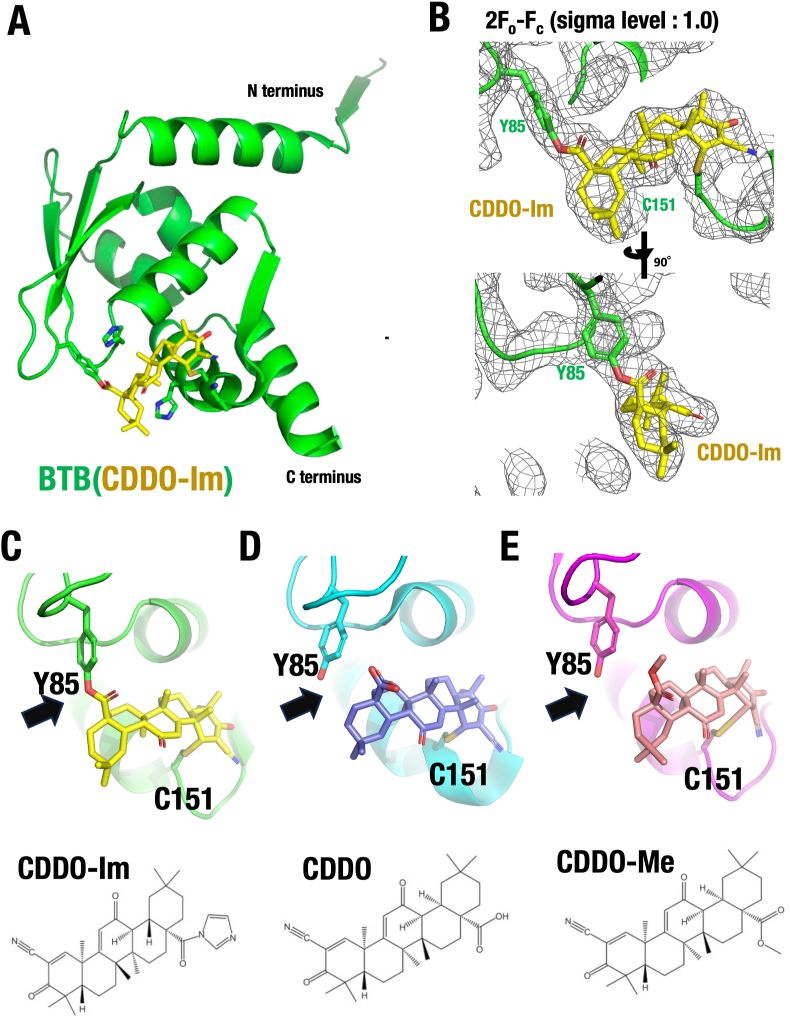
**Co-crystal structure of KEAP1-BTB with CDDO-Im.** (**A**) Co-crystal structure of KEAP1-BTB monomer (green) bound to CDDO-Im (yellow). (**B**) Electron density map 2F_o_-F_c_ (sigma level 1.0) showing CDDO-Im (yellow) and the surrounding residues Cys151 and Tyr85 (green). (**C-E**) Binding configurations of KEAP1-BTB with CDDO-Im (**C**), CDDO-Me (**D**) and CDDO (**E**). Note that only CDDO-Im forms a crosslink between Cy151 and Tyr85, whereas CDDO and CDDO-Me bind exclusively to Cys151. Structure: CDDO-Im-modified BTB (PDB 9UJI), CDDO-modified BTB (PDB 4CXT) and CDDO-Me-modified BTB (PDB 9UJJ).

Interestingly, mutational analyses of KEAP1 have highlighted the importance of the Cys151 residue for the CDDO-Im-mediated NRF2 activation [15,17,18,50]. However, mass spectrometric analyses failed to detect covalent modification of Cys151 by CDDO-Im [51]. Therefore, we investigated how CDDO-Im modifies the KEAP1-BTB domain using a co-crystal structural approach. We successfully determined the co-crystal structure of the mouse KEAP1-BTB domain bound to CDDO-Im at 2.4 Å resolution (Fig. 2A). This analysis led us to a key discovery: as shown in the electron density maps in Fig. 2B and Supplementary Fig. S2, CDDO-Im forms simultaneous interactions with both Cys151 and Tyr85 within the BTB domain. The interaction with Tyr85 involves the imidazole moiety of CDDO-Im (Supplementary Fig. S3). This finding is consistent with previous mass spectrometric data that identified Tyr85, but not Cys151, as a modification site [51]. These results clearly indicates that CDDO-Im forms a cross-link between Cys151 and Tyr85 in the KEAP1-BTB domain.

**Cross-linking of Cys151 and Tyr85 is unique to CDDO-Im.** Since compounds in the CDDO family exhibit the strongest NRF2-inducing activity among BTB domain-interacting chemicals (Class I inducers) [6], we sought to determine whether the cross-link between Cys151 and Tyr85 is a common feature among CDDO family compounds. To investigate this, we compared the binding modes of CDDO-Im (Fig. 2C) and CDDO (Fig. 2D) to the BTB domain. We utilized the previously reported co-crystal structure of the BTB domain with CDDO (PDB ID: 4CXT) [25]. Comparison of both two co-crystal structures revealed that the positioning of CDDO-Im and CDDO within the BTB domain is very similar, and both compounds covalently bind to Cys151. However, binding to Tyr85 was observed only in the CDDO-Im structure (Fig. 2C) and not in the case of CDDO (Fig. 2D; see black arrow), suggesting that Tyr85 modification is unique to CDDO-Im.

To further test whether the Cys151-Tyr85 crosslink is specific to CDDO-Im or shared by other members of the CDDO family, we analyzed another derivative, CDDO-methyl ester (CDDO-Me or Bardoxolone methyl®), which has advanced to phase 3 clinical trials for diabetic nephropathy [22]. CDDO-Me was co-crystalized with the mouse KEAP1-BTB domain and its structure was resolved at 3.4 Å resolution (Fig. 2E, Supplementary Fig. S4). We found that CDDO-Me binds to Cys151 but not Tyr85, indicating that Tyr85 modification in KEAP1 is unique to CDDO-Im. Based on the chemical structure of these compounds, the imidazole moiety of CDDO-Im likely accounts for its ability to modify Tyr85 (Supplementary Fig. S3). Collectively, these results demonstrate that CDDO family compounds consistently modify Cys151 in the KEAP1 BTB domain. They also suggest that the pocket structure accommodating both Cys151 and Tyr85 is critical for the sensor function of the KEAP1-BTB domain.

**Fig. 3:**
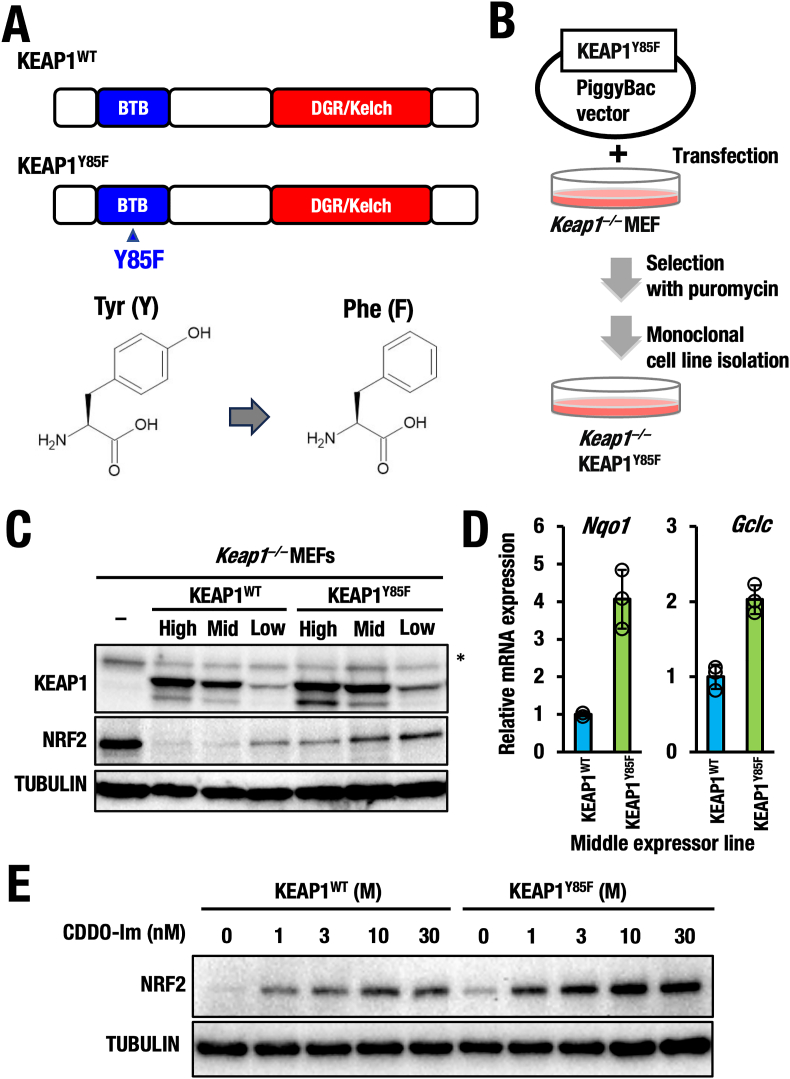
**KEAP1-Tyr85 is functionally important for homeostatic KEAP1 activity. (A)** KEAP1^WT^ and KEAP1^Y85F^ mutant. Note that the difference of tyrosine and phenylalanine is presence or absence of hydroxy group at side chain of amino acid residue. **(B)** Generation of stable cell lines expressing KEAP1^Y85F^ mutant using *Keap1*^*−/−*^ MEF cells by selection with puromycin and monoclonal cell line isolation. (**C**) Protein levels of KEAP1 and NRF2 in high, middle or low expressor cell lines of KEAP1^WT^ or KEAP1^Y85F^ mutant. (**D**) Relative mRNA levels of *Nqo1* or *Gclc* genes in the middle expressor cell lines of KEAP1^WT^ or KEAP1^Y85F^ mutant. (**E**) NRF2 protein levels of in middle expressor cell lines of KEAP1^WT^ or KEAP1^Y85F^ mutant treated with 0, 1, 3, 10, and 30 μM of CDDO-Im for 3 h.

**Tyr85 of the KEAP1-BTB domain is important for regulating basal NRF2 activity.** Our discovery that CDDO-Im forms a crosslink between Tyr85 and Cys151 in KEAP1 suggests that Tyr85 is located in close proximity to Cys151 and may influence the function of this key sensor residue. To assess the functional importance of Tyr85, we introduced a substitution mutation replacing tyrosine with phenylalanine (Y85F), which differs only by the presence or absence of a hydroxy (-OH) group (Fig. 3A).

To eliminate the influence of endogenous KEAP1 [15,16], we generated stable cell lines expressing either wild-type KEAP1 (KEAP1^WT^) or the Y85F mutant (KEAP1^Y85F^) in *Keap1*^*−/−*^ mouse embryonic fibroblasts (MEFs) using the PiggyBac transposon system (Fig. 3B). We established sets of cell lines expressing high, medium, and low levels of either KEAP1^WT^ and KEAP1^Y85F^ mutant and compared their NRF2 protein levels. As expected, NRF2 protein levels decreased incrementally with increasing levels of KEAP1^WT^ expression. A similar trend was observed with KEAP1^Y85F^-expressing cells (Fig. 3C). However, a notable finding was that the KEAP1^Y85F^-expressing cells consistently exhibited significantly higher NRF2 protein levels than their KEAP1^WT^ counterparts. This suggests that the basal activity of KEAP1^Y85F^ to degrade NRF2 is reduced compared to KEAP1^WT^.

Supporting this contention, transcript levels of *Nqo1* and *Gclc*, two well-characterized NRF2 target genes, were significantly elevated in cells expressing KEAP1^Y85F^ compared to those expressing KEAP1^WT^, particularly at the medium expression level (Fig. 3D). These results indicate that the hydroxy group of Tyr85 is crucial for the basal activity of KEAP1 in promoting NRF2 degradation.

To evaluate the role of Tyr85 in KEAP1's recognition of CDDO-Im, we treated mid-level expressor cells of both KEAP1^WT^ and KEAP1^Y85F^ with CDDO-Im (Fig. 3E). Consistent with previous results, the basal level of NRF2 protein was higher in KEAP1^Y85F^-expressing cells compared to KEAP1^WT^**-**expressing cells. Importantly, both cell types showed dose-dependent accumulation of NRF2 protein upon CDDO-Im treatment. Notably, the accumulation of NRF2 protein was more pronounced in KEAP1^Y85F^ -expressing mutant cells than those in KEAP1^WT^ cells. These findings support the contention that Tyr85 contributes to the regulation of NRF2 levels moderately that can be clearly seen in unstimulated conditions, but the contribution becomes unnoticeable under CDDO-Im-mediated NRF2 induction. In contrast, Cys151 in the KEAP1 BTB domain is essential for CDDO-Im-induced NRF2 activation [15,17,18].

**Fig. 4:**
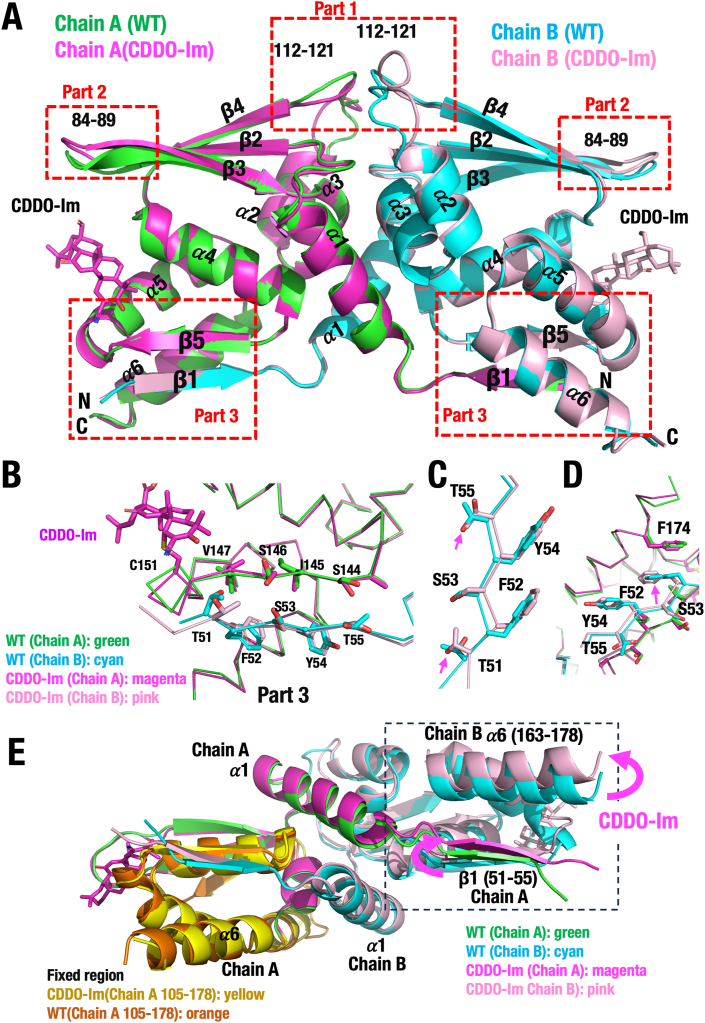
**Structural changes of the KEAP1-BTB domain by CDDO-Im modification. (A)** Superimposed structure of the KEAP1-BTB homodimer with CDDO-Im (PDB 9UJI) comparing the apo form of the BTB dimer (PDB 9UJG). Note that CDDO-Im modification provoked changes in three parts in the BTB domain, Part 1 (loop 112–121), Part 2 (loop 84–89) and Part 3 (β1 (strand 51–55)/β5(strand 144–147)/α6 (helix 163–178)). **(B**–**D)** Superimpose structures of Part 3 (β1 (strand 51–55)/β5 strand 144–147)/α6 (helix 163–178)) of the BTB-WT, and CDDO-Im-modified BTB. (**B**) β5 of Chain A and β1 of Chain B with CDDO-Im. (**C**) β1 of Chain B. (**D**) β1 of Chain B and F174 of Chain A. Arrows indicate changes brought by CDDO-Im binding. (**E**) Fixation of apo-BTB and CDDO-Im-BTB of the Chain A CUL3-intearacting region provokes marked changes in the orientations of the β1 (strand 51–55) of Chain A and α6 (helix 163–178) of Chain B. When apo-BTB (green) and CDDO-Im-BTB (magenta) of the Chain A CUL3-intearacting region (105–178) are fixed, the orientations of the β1 (strand 51–55) (green and magenta) of Chain A and α6 (helix 163–178) (cyan and pink) of Chain B structures in the dimer was changed (red arrows) significantly. BTB-apo (PDB 9UJG) and CDDO-Im-modified BTB (PDB 9UJI).

**Structural changes in the BTB domain induced by CDDO modification**. Modification of KEAP1-Cys151 by CDDO compounds has been identified as a common mechanism regulating its E3 ubiquitin ligase activity. A key question, therefore, is how this modification affects the structure of the BTB domain. To address this, we closely examined the BTB dimer structure in the presence and absence of CDDO-Im (Fig. 4A). As indicated as dotted boxes in Fig. 4A, CDDO-Im modification induced structural changes in three parts of the BTB dimer: (Part 1) loop 112–121, (Part 2) loop 84–89, and (Part 3) the regions encompassing strands 51–55 (β1), 144–147 (β5) and 163–178 (α6).

Loop 112–121 (Part 1) and Gln163 of KEAP1 correspond to the region known to interact with CUL3, as reported in the KEAP1-CUL3 complex structure (PDB ID 5NLB) [52]. These regions, including loop 112–121 and helix α6 (163–178), are highlighted in orange in Supplementary Fig. S5A. Notably, loop 112–121 showed considerable variability among the eight molecules in the asymmetric unit of the CDDO-Im-modified BTB structure (Supplementary Fig. S5B), suggesting that this loop is unlikely to be the primary cause of the CDDO-Im-induced destabilization of the KEAP1-CUL3 complex.

Structural changes in loop 84–89 (Part 2) were observed when comparing the apo form of the BTB dimer with the CDDO-Im-modified BTB dimer (Supplementary Fig. S5C). This structural alteration, elicited by CDDO-Im, was not observed in the BTB domain co-crystalized with CDDO (PDB ID: 4CXT) [25] (Supplementary Fig. S5D). This finding suggests that CDDO-Im, but not CDDO, induces a structural distortion in loop 84–89 by forming a cross-link between Cys151 and Tyr85 in the KEAP1. Since structural changes in both loop 112–121 (Part 1) and loop 84–89 (Part 2) are observed only in the presence of CDDO-Im, and not with CDDO, we conclude that these structural alterations are unique to CDDO-Im. Therefore, they are unlikely to represent a common mechanism by which the CDDO family of compounds destabilizes the KEAP1-CUL3 complex.

**Identification of the structural motif in the BTB homodimer responsible for reduction of KEAP1 ubiquitin ligase activity by Cys151 modification.** Multiple functional studies have shown that the CDDO family compounds reduce the KEAP1 ubiquitin ligase activity through modification of Cys151 [[15], [17], [18], [50]]. We hypothesized that structural changes commonly induced by CDDO compounds in the BTB homodimer are responsible for this Cys151-mediated inactivation of KEAP1. Through detailed comparison of the apo-form and the CDDO-Im-bound BTB homodimer, we identified three motifs in the BTB domain that undergo structural changes upon CDDO-Im binding. As discussed above, two of three motifs are unlikely to be functionally relevant. Therefore, we focused on the third motif, referred to as Part 3, which consists of strands 51–55 (β1), 144–147 (β5), and 163–178 (α6).

As shown in Fig. 4B, Part 3 is formed by an antiparallel β-sheet between strands 51–55 (β1) of Chain B (shown in cyan) and 144–147 (β5) of Chain A (green), located near helix 163–178 (α6) of Chain A. A similar structure is also formed in the reciprocal part of the homodimer, with Chain A and B switching roles. To assess the effect of CDDO-Im, we compared the Part 3 structure in the apo and CDDO-Im-modified BTB homodimers. As shown in Fig. 4B–D, CDDO-Im induces slight conformational shifts in strands β1 and β5. In particular, the side chain orientations of Thr51 and Thr55 (Fig. 4C) and Phe52 (Fig. 4D) appear to be altered.

Although these local conformational changes may seem minor, we hypothesized that structural alterations in one BTB monomer may influence the spatial arrangement of the opposing monomer in the homodimer, potentially affecting CUL3 binding. To test this, we compared the CUL3-binding regions (residues 105–178) of the apo and CDDO-Im–bound BTB dimers. As shown in Fig. 4E, when the CUL3-binding region of Chain A (residues 105–178) is aligned between apo-BTB (green) and CDDO-Im–modified BTB (magenta), noticeable shifts are observed in strand β1 (green vs. magenta) of Chain A and helix α6 (cyan vs. pink) of Chain B (highlighted with red arrows). These findings indicate that CDDO-Im induces significant structural changes in Part 3 of the BTB homodimer, while changes within a single BTB monomer are relatively small and insufficient to explain KEAP1 inactivation. Therefore, we propose that the modification of Cys151 by CDDO-Im causes structural rearrangement within the BTB dimer that weakens CUL3 interaction, contributing to NRF2 activation.

**Fig. 5:**
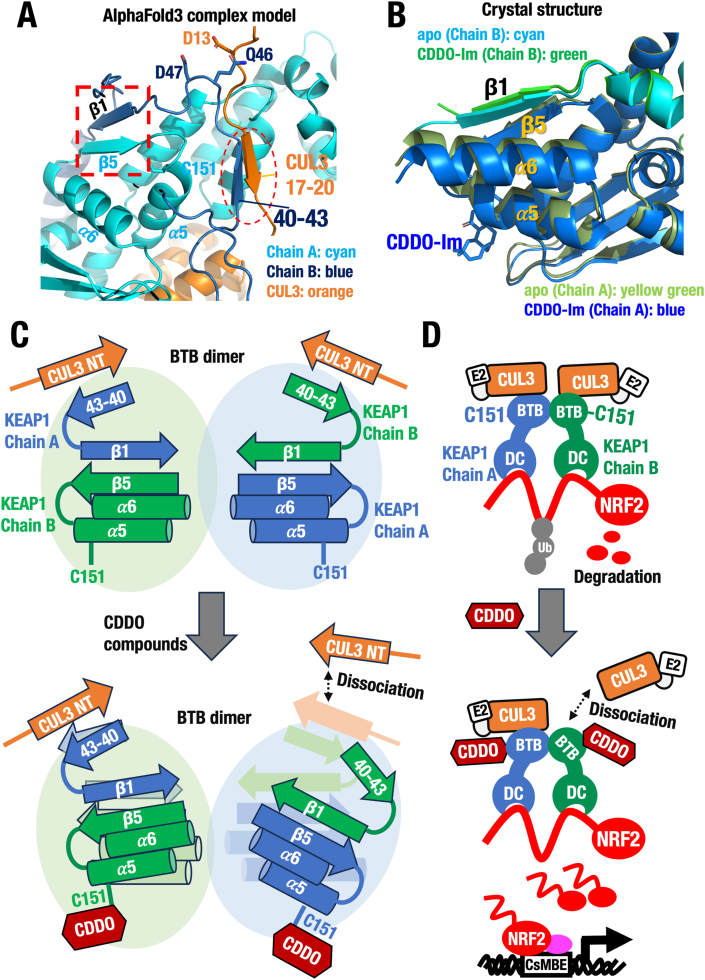
**CDDO-Im-modification dissociates one CUL3 molecule from the KEAP1 homodimer complex.** (**A**) AlphaFold3 complex model for the KEAP1-Cys151 surrounding region and N-terminus region of CUL3 (CUL3 NT). Note that sheet 40–43 connecting to β1 (sheet 51–55) of KEAP1 Chain B (blue) interacts with the CUL3 NT (orange). (**B**) Superimposed structure of β5 (144–151) and β1 (51–55) in the KEAP1-BTB domain of WT apo (PDB 9UJG, cyan and yellow green) and CDDO-Im-modified WT (PDB 9UJI, green and blue) structures. (**C**, **D**) A model proposing the molecular mechanism how CDDO compounds introduce changes affecting the affinity of the KEAP1-CUL3 complex and activate the NRF2 pathway via the KEAP1-Cys151 sensor. (**C**) Structural basis for the dissociation of one CUL3 molecule from the KEAP1 homodimer, specially focusing the structural changes in the BTB domain. (**D**) Influences of one CUL3 molecule dissociation to the entire KEAP1-CUL3 complex activity. Note that the one CUL3 molecule dissociation makes the ubiquitin ligase activity substantially inactive.

**CDDO-mediated alteration of the BTB homodimer reduces KEAP1–CUL3 interaction.** It has been reported that the N-terminal region of CUL3 (residues 1–22), referred to as CUL3-NT, is required for its interaction with KEAP1 [52]. The KEAP1–CUL3 complex model derived from AlphaFold3 further supports this, showing that the side chain of KEAP1 Gln46 is positioned to interact with CUL3 Asp13 (Fig. 5A), indicating direct contact between CUL3-NT and KEAP1. Additionally, the AlphaFold3 model reveals that KEAP1 residues 40–43 form a β-sheet with residues 17–20 of CUL3-NT (Fig. 5A), contributing to KEAP1–CUL3 heterodimer formation. Interestingly, the same modeling approach also indicates that strand 51–55 (β1) of KEAP1 Chain B forms a swapped β-sheet with strand 144–147 (β5) of Chain A within the BTB domain dimer.

This is particularly relevant in light of our structural analysis, which showed that CDDO-Im induces significant conformational changes in Part 3 of the BTB homodimer, specifically involving strands 51–55 (β1) and 144–147 (β5) (see Fig. 4A). Initially, we were puzzled by the fact that CDDO-Im causes only minimal structural changes within a single BTB monomer, which seemed insufficient to explain KEAP1 inactivation. However, upon further analysis, we found that the CDDO-Im-induced changes in Part 3 of the homodimer also affect the conformation of strand 40–43 in Chain B.

Based on this, we propose that CDDO-Im binding disrupts the interaction between strand 40–43 of the BTB domain and strand 17–20 of CUL3-NT (Fig. 5A). Supporting this idea, our BTB–CDDO-Im co-crystal structure shows that Cys151 modification leads to a shift in helix 149–162 (α5), followed by conformational changes in strand 144–147 (β5) of Chain A and strand 51–55 (β1) of Chain B (Fig. 5B).

Given the structural relationship between strand 51–55 (β1) and strand 17–20 of CUL3-NT, it is highly plausible that the change in β1 triggers alterations in strand 40–43 of Chain B, thereby weakening the KEAP1–CUL3 interaction. This cascade of conformational changes is summarized in Fig. 5C. Notably, as illustrated in Fig. 5D, we hypothesize that these CDDO-Im-induced spatial rearrangements in the BTB homodimer ultimately lead to the dissociation of one CUL3 molecule from the KEAP1 homodimer.

**Fig. 6:**
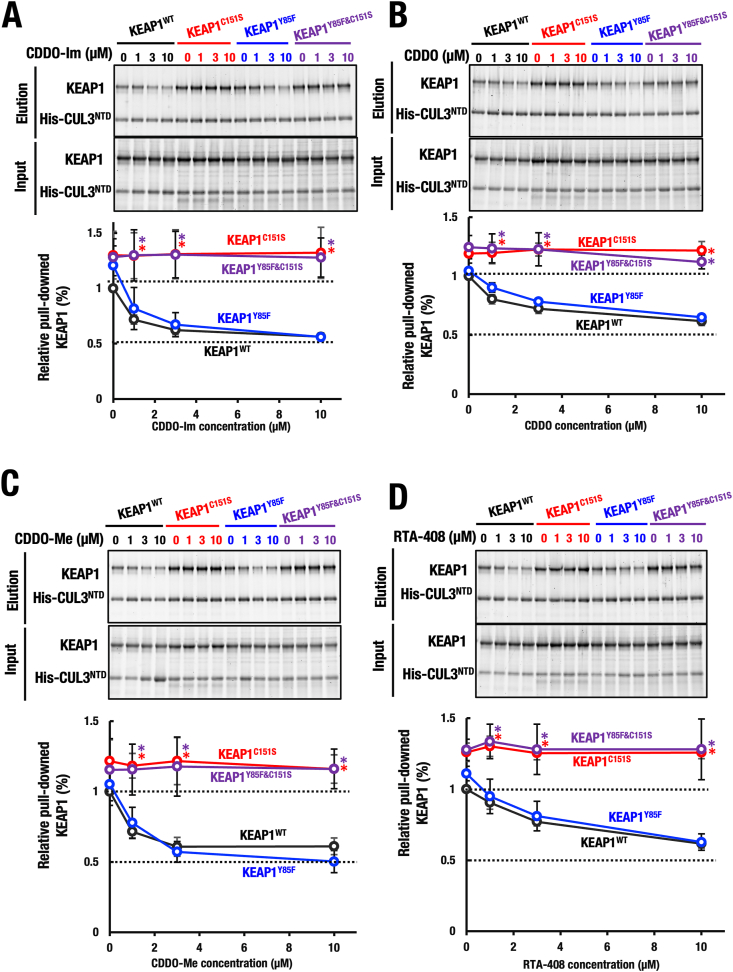
**CDDO derivatives commonly inhibit the KEAP1-CUL3 interaction in Cys151-dependent and Tyr85-independent manners.** Recombinant KEAP1 proteins KEAP1^WT^, KEAP1^C151S^, KEAP1^Y85F^, and KEAP1^C151S&Y85F^ were incubated with 0, 1, 3, or 10 μM of CDDO-Im (**A**), CDDO (**B**), CDDO-Me (**C**) or RTA-408 (**D**). These KEAP1 and derivative proteins were mixed with recombinant His-CUL3^NTD^ protein. After incubation, these proteins were pull-downed with Ni-NTA magnetic agarose beads, and the eluted proteins were electrophoresed and stained by oriole. The pull-downed KEAP1 intensities were measured, and the average values were presented (n = 3, Mean ± SD). One-way ANOVA followed by Dunnett's test vs WT. ∗p < 0.05. Note that these electrophiles abrogated His-CUL3^NTD^ protein binding to KEAP1^WT^ and KEAP1^Y85F^ to approximately half levels of electrophile-free control level, while His-CUL3^NTD^ protein binding to KEAP1^C151S^ and KEAP1^C151S&Y85F^ was not affected by the electrophiles.

**The KEAP1-CUL3 interaction is reduced to the approximately half by CDDO compound treatment.** To test the hypothesis that one CUL3 molecule dissociates from the KEAP1 homodimer upon CDDO treatment, we performed pull-down experiments using full-length KEAP1 and the N-terminal domain of CUL3 (CUL3-NTD), which interacts with the KEAP1 BTB domain [4,10]. As shown in Fig. 6A, CDDO-Im treatment reduced the interaction of KEAP1^WT^ and CUL3^NTD^ to approximately 50 %. In contrast, the KEAP1^C151S^-CUL3 interaction was unaffected by CDDO-Im, further supporting the critical role of Cys151 in this interaction reduction. Intriguingly, the interaction between KEAP1^Y85F^ and CUL3^NTD^ was also reduced by CDDO-Im, but the reduction was less pronounced compared to the case for KEAP1^WT^ and CUL3^NTD^, suggesting that Tyr85 partially influences on the sensitivity of Cys151 (Fig. 6A, blue bars). Conversely, the interaction between the double mutant KEAP1^Y85F&C151S^ and CUL3 remained unchanged upon CDDO-Im treatment (purple bars).

Co-crystal structure analysis had showed that both CDDO and CDDO-Me bind exclusively to Cys151, not Tyr85 (Fig. 2). To determine whether these structural insights align with functional outcomes, we assessed the effects of CDDO and CDDO-Me on KEAP1–CUL3 interactions (Fig. 6B and C). Similar to CDDO-Im, both CDDO and CDDO-Me reduced the interactions of KEAP1^WT^ and the KEAP1^Y85F^ with CUL3^NTD^ (black and blue bars, respectively), but had no effect on interactions involving KEAP1^C151S^ or KEAP1^Y85F&C151S^ (red and purple bars, respectively).

To further confirm this trend, we tested another CDDO family compound, RTA-408 (Omaveloxolone), approved for the treatment of Friedreich's ataxia [23]. RTA-408 also reduced the KEAP1^WT^-CUL3^NTD^ and KEAP1^Y85F^-CUL3 interactions (Fig. 6D, black and blue bars), but had no effect on KEAP1^C151S^-CUL3 or KEAP1^Y85F&C151S^-CUL3 interactions (red and purple bars).

These results collectively demonstrate a common mechanism among CDDO family compounds—including CDDO-Im, CDDO, CDDO-Me, and RTA-408—where Cys151 modification reduces KEAP1–CUL3 interaction, while Tyr85 is not involved. We conclude that this Cys151-dependent mechanism weakens the KEAP1 ubiquitin ligase activity by decreasing KEAP1–CUL3 complex stability to approximately half, likely through the dissociation of one CUL3 molecule from the KEAP1 homodimer, as supported by our structural and biochemical data.

**Fig. 7:**
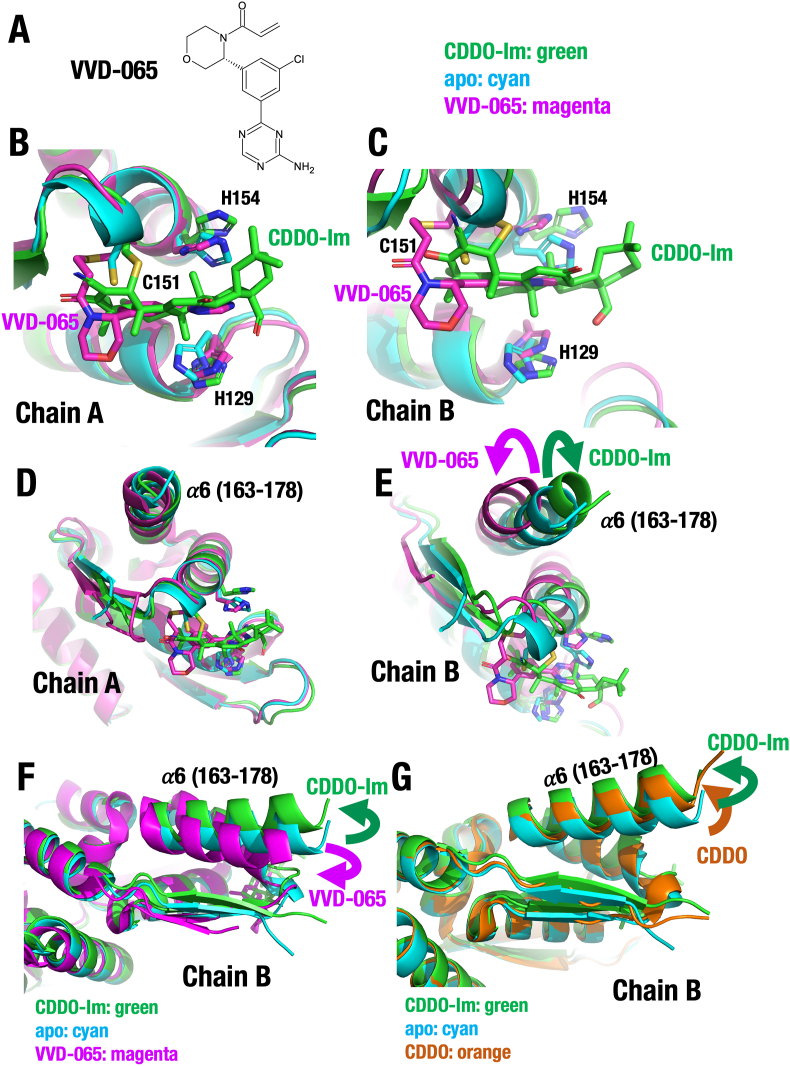
**KEAP1 activator VVD-065 introduces changes in the BTB homodimer to the orientation opposite to those of KEAP1 inhibitor CDDO-Im. (A)** Chemical structure of VVD-065. (**B**–**F**) Superimposed structures of the apo BTB (PDB 9UJG, cyan), CDDO-Im-modified BTB (PDB 9UJI, green) and VVD-065-modified BTB (PDB 9DU7, magenta) domains. (**B, C**) The Cys151-surronding region of Chain A (**B**) and Chain B (**C**). (**D, E**) Helix 163–178 of Chain A (**D**) and Chain B (**E**). (**F)** Superimposed structures of helix 163–178 of Chain B of the apo BTB (cyan), CDDO-Im-modified BTB (green) and VVD-065-modified BTB (magenta) domains. Note that KEAP1 activator VVD-065 introduces changes in the BTB homodimer to the orientation opposite to those of KEAP1 inhibitor CDDO-Im. Violet arrows for VVD-065 and green arrows for CDDO-Im. **(G**) Superimposed structures of helix 163–178 of Chain B of the apo BTB (PDB 9UJG, cyan), CDDO-Im-modified BTB (PDB 9UJI, green) and CDDO-modified BTB (PDB 4CXT, orange) domains. Note that bindings of CDDO-Im and CDDO introduces changes toward the same direction. Green arrow for CDDO-Im and orange arrow for CDDO.

**KEAP1 activator VVD-065 alters the BTB homodimer structure in the opposite direction of KEAP1 inhibitor CDDOs.** VVD-065 is a recently developed KEAP1 activator (*i.e.,* NRF2 inhibitor) that binds to the BTB domain and acts in a Cys151-dependent manner [53] (PDB ID: 9DU7) (Fig. 7A). Like CDDO compounds, VVD-065 modifies Cys151; however, unlike CDDOs, it enhances the ubiquitin ligase activity of KEAP1, suggesting a fundamentally different mechanism. We therefore hypothesized that VVD-065 induces distinct structural changes in the BTB homodimer compared to those observed with CDDO compounds.

To test this, we compared the BTB homodimer structures determined in this study with that of the VVD-065–BTB homodimer (PDB ID: 9DU7). As with the analysis of the CDDO-Im–modified BTB structure, we aligned Chain A of the CUL3-interacting region (residues 105–178) of the apo-BTB and VVD-065–BTB dimers (Fig. 7B–F), and superimposed the structures of the VVD-065–BTB homodimer (magenta) and CDDO-Im–modified BTB homodimer (green). Like CDDO-Im, VVD-065 modifies Cys151 of KEAP1 (Fig. 7B and C). Notably, VVD-065 enters the Cys151-binding pocket in a manner distinct from the CDDO compounds.

As expected, both VVD-065 and CDDO-Im modifications induce only minor conformational changes in Chain A (*i.e.,* one monomer) of the BTB homodimer (Fig. 7D). In contrast, Chain B (the opposite side of the BTB dimer) undergoes a significant reorientation of helix 163–178 (α6) upon binding to either VVD-065 or CDDO-Im, as shown in the superimposed images in Fig. 7E. Interestingly, while VVD-065 shifts Chain B in one direction compared to apo-BTB, CDDO-Im shifts it in the opposite direction (Fig. 7E and F). The reorientation of helix 163–178 (α6) observed with CDDO-Im is consistent and occurs in the same direction in the structure of CDDO-bound KEAP1 (PDB ID: 4CXT) (Fig. 7G).

**Fig. 8:**
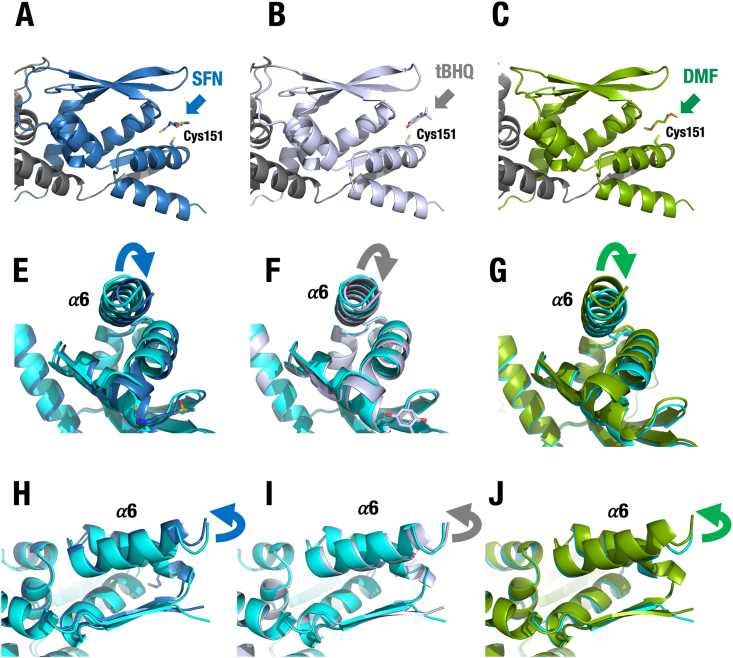
**Molecular docking models of the BTB domain complexed with Cys151-targeting NRF2 inducers.** AlphaFold3 was utilized for generation of the BTB complexed with SFN (**A**, **E** and **H**; blue), tBHQ (**B**, **F** and **I**; gray) and DMF (**C**, **G** and **J**; green). (**A-C**) Overall views of the KEAP1-BTB complex bound to SFN (**A**), tBHQ (**B**) and DMF (**C**) (indicated by arrows). (**E**–**J**) Superimposed models of helix 163–178 of Chain B of the apo BTB (cyan) with each compound. Note that all three compounds induce conformational changes in the BTB homodimer similar in orientation to those caused by the KEAP1 inhibitor CDDOs.

To further provide mechanistic insight into the other Cys151-targeting NRF2 inducers, we generated the molecular docking models of the BTB complexed with SFN, tBHQ and DMF, which are classified into Class I; Cys151-prefering NRF2 inducers [15]. We utilized the AlphaFold3 to ask whether these chemicals induce structural changes in Part3 of the BTB homodimer as the cases for CDDO and VVD compounds. We found that, although slighter than the case of CDDO compounds, all the three NRF2-inducing chemicals induce the structure change to the same orientation as the case of CDDO compounds (Fig. 8). Especially, the effects of SFN are stronger than the two others, which is correlated with their potency of NRF2-inducing activity. In addition, the structural changes induced by SFN, tBHQ and DMF appear to be smaller than those caused by CDDO compounds, supporting the notion that their inhibition of the KEAP1-CUL3 interaction is limited [10]. These results strongly support the conclusion that the Cys151-targeting NRF2 inducers operate common mechanisms to inhibit the ubiquitination of NRF2.

**Fig. 9:**
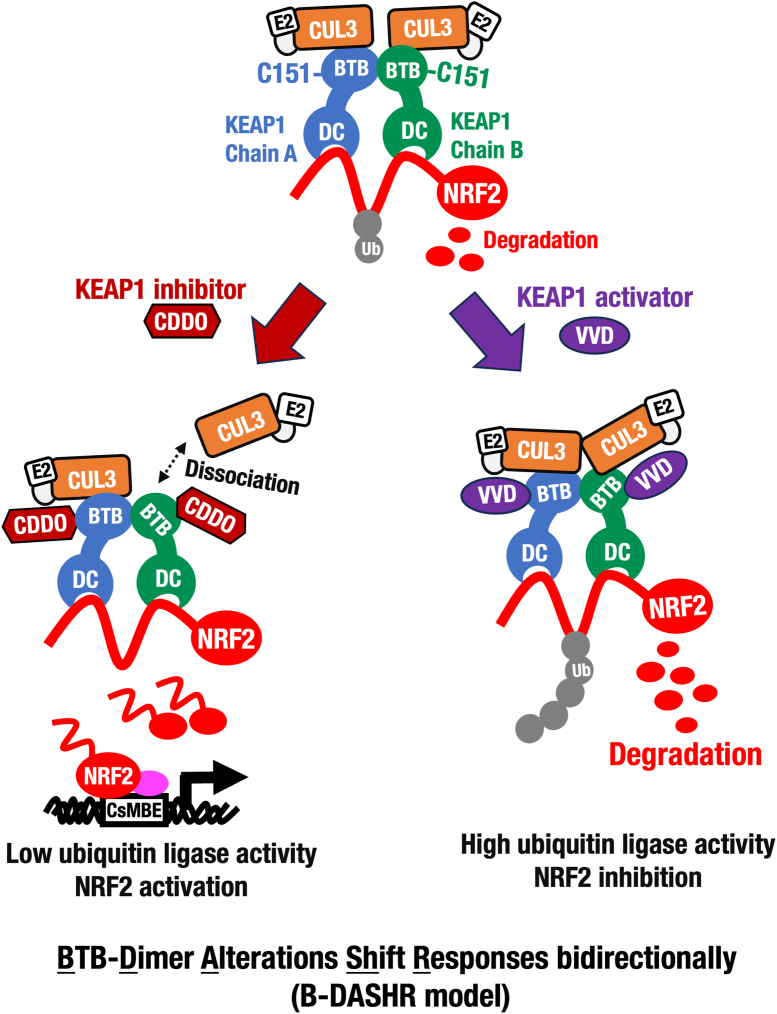
**The B-DASHR (BTB-Dimer Alterations Shift Responses Bidirectionally) model for the KEAP1 sensor regulation.** KEAP1 activator VVD-065 changes structure of the BTB homodimer to the opposite direction to that of the KEAP1 inhibitor CDDO compounds, unequivocally demonstrating that the BTB dimer structural alterations are the key regulations of the KEAP1-CUL3 ubiquitin ligase activity to both activating and inhibiting the NRF2 activity.

As shown in Fig. 9, this study demonstrates that structural changes in the KEAP1-BTB homodimer induced by CDDO family compounds lead to dissociation of one CUL3 molecule from the KEAP1 dimer, thereby reducing KEAP1-CUL3 ubiquitin ligase activity. Conversely, VVD-065 induces BTB dimer changes in the opposite direction, which correlate with increased KEAP1-CUL3 ubiquitin ligase activity. Thus, KEAP1 inhibitors (*e.g.,* CDDO compounds) and the KEAP1 activator (VVD-065) modulate BTB dimer structure in opposite directions. This study reveals the molecular basis by which the KEAP1-CUL3 ubiquitin ligase activity is finely regulated through structural modifications of the BTB domain upon electrophile binding.

## 4 Discussion

In this study, we sought to elucidate the molecular mechanisms by which electrophilic stress regulates the ubiquitin ligase activity of KEAP1 through binding to stress-sensor cysteine residues. Using a combination of co-crystal structure analysis and functional studies of the KEAP1 BTB domain, we revealed that KEAP1 contains an elaborately designed sensor site that modulates its ubiquitin ligase activity in response to electrophilic compounds. Both NRF2 activators and inhibitors target Cys151 and influence the KEAP1-CUL3 complex by altering the spatial arrangement of its CUL3-binding sites. Notably, these activators and inhibitors induce structural shifts in the BTB homodimer in opposite directions.

**Spatial environment in the KEAP1-BTB domain maintains Cys151-based sensor activity.** In this study, we provide compelling evidence that CDDO family compounds, including CDDO, CDDO-Im, CDDO-Me, and Omaveloxolone, which are either clinically approved or in development, commonly bind to Cys151 in the KEAP1 BTB domain. Our co-crystal structure analysis revealed that CDDO-Im forms a cross-link between Cys151 and Tyr85 in KEAP1, whereas other CDDO family compounds, such as CDDO and CDDO-Me, do not. Subsequent functional analyses demonstrated that Tyr85 is essential for maintaining Cys151 reactivity under basal, unstressed conditions. Intriguingly, we also found that the KEAP1-Y85F mutant retains the ability to sense CDDO-Im, indicating that the hydroxyl group of Tyr85, and thus the Cys151-Tyr85 cross-link, is dispensable for sensing CDDO-Im and for the electrophile-induced inactivation of KEAP1's ubiquitin ligase activity.

We hypothesize that Tyr85 plays a key role in enabling the BTB domain to sense weak endogenous electrophiles, such as fumarate, itaconate, and other metabolites from glycolysis and the TCA cycle, that are constitutively present in cells. In contrast, strong electrophilic inducers like CDDO compounds can modify Cys151 with sufficient potency even in the absence of Tyr85. We propose that Tyr85 contributes to Cys151 reactivity and the spatial organization of the sensor site by influencing surrounding residues, including His129 and His154. These residues are highly conserved among vertebrates (Supplementary Fig. S6), suggesting that the spatial configuration of the Cys151 sensor is both functionally and evolutionarily important for maintaining its stable sensing capacity. The molecular mechanisms that differentiate strong NRF2 inducers from weak constitutive inducers remain to be elucidated.

**Cross-link formation between Cys151 and surrounding amino acid residues**. Another example of cross-link formation within the KEAP1-BTB domain has been previously reported: methylglyoxal modifies KEAP1 by forming a methylimidazole cross-link between Cys151 and Arg135, known as MICA (see underlines) [54]. In this study, we discovered a CDDO-Im-mediated cross-link formation between Cys151 and Tyr85 in KEAP1. Our crystal structure data show that Cys151 is surrounded by basic amino acid residues, including His129 and His154, within the BTB domain, suggesting the possibility that other electrophiles may also induce cross-links between Cys151 and neighboring residues. However, our current analysis demonstrates that while the spatial arrangement of Cys151 is critically important, cross-link formation within the BTB domain may not be essential for KEAP1's electrophile-sensing mechanism.

**Molecular mechanisms by which the KEAP1-BTB domain contributes to NRF2 ubiquitination.** One of the key findings from our co-crystal structure analyses is that electrophilic modification of Cys151 induces structural changes in the KEAP1-BTB homodimer. While previous seminal studies have shown that CDDO modification of Cys151 weakens the KEAP1–CUL3 interaction [10,25], the molecular mechanisms by which CDDO-Im affects this interaction have remained unclear. In this study, we propose a novel concept based on structural changes in the BTB homodimer. This concept emerges from detailed comparisons of the homodimer structures in the presence and absence of electrophiles—analyses that cannot be achieved by examining the BTB monomer alone. This approach provides new insights into how electrophiles regulate KEAP1–CUL3 ubiquitin ligase activity.

We found that CDDO-Im binding and modification of Cys151 induce substantial structural changes in the BTB homodimer, which in turn disrupt the interaction between CUL3-NT and KEAP1-BTB, thereby weakening the KEAP1–CUL3 interaction. A series of structural and biochemical analyses support this model. Biochemical studies have shown that CUL3-NT is crucial for the interaction between KEAP1 and CUL3 [52]. Co-crystal structure analyses of the KLHL11–CUL3 complex indicate that CUL3-NT is essential for binding BTB-containing proteins [55]. Additionally, cryo-EM analyses of the KLHL12–CUL3 complex show that CUL3-NT participates in complex assembly and stoichiometry [56]. Together, these findings strongly support the idea that interaction between the CUL3-NT and the BTB domain is a critical determinant of ubiquitin ligase activity across BTB-containing proteins.

Our structural analyses reveal that this CUL3-NT–BTB interaction serves as a key regulatory point for controlling NRF2 activity in response to electrophiles. Given that the KEAP1–CUL3 interaction is among the weakest compared to other BTB-containing protein–CUL3 interactions [57], this relatively low affinity allows for fine-tuning of the NRF2 ubiquitination process via subtle structural changes in the BTB dimer induced by electrophilic modification of Cys151.

**Alterations of the BTB dimer structure bidirectionally shift cytoprotective responses.** Another key finding of this study is that the KEAP1 activator VVD-065 shifts the BTB homodimer structure in the opposite direction to that induced by KEAP1 inhibitors, such as the CDDO compounds. This observation led us to propose an intriguing hypothesis: that structural alterations in the BTB homodimer represent a central regulatory mechanism for KEAP1–CUL3 ubiquitin ligase activity, capable of modulating NRF2 activity in both activating and inhibiting directions (Fig. 8). To describe this concept, we introduce the term BTB-dimer Alterations Shift Responses bidirectionally (B-DASHR) model. The B-DASHR mechanism illustrates how KEAP1 serves as a sensor, regulating NRF2 activity by responding to both activating and inhibiting compounds that influence KEAP1–CUL3-mediated ubiquitin ligase activity. We believe that the insights gained from this study offer a novel framework for the development of therapeutic agents targeting the KEAP1-BTB domain. For instance, molecular docking of additional VVD analogs beyond VVD-065 could guide future development of KEAP1 activators.

In summary, this study addressed fundamental questions regarding the structural and mechanistic basis by which KEAP1 functions as an elaborate sensor for NRF2-inducing chemicals. Our structure analysis revealed that the Cys151-centered electrophile sensor within the BTB domain homodimer operates in a local environment that maintains the reactive thiol state of Cys151. Both activators and inhibitors of NRF2 target Cys151 and modulate the ubiquitin ligase activity of the KEAP1-CUL3 complex by altering the spatial arrangement of CUL3-binding sites. Intriguingly, these activators and inhibitors shift the BTB homodimer structure in opposite directions.

## CRediT authorship contribution statement

**Takafumi Suzuki:** Writing – review & editing, Writing – original draft, Supervision, Investigation, Funding acquisition, Conceptualization. **Kenji Takagi:** Writing – review & editing, Writing – original draft, Visualization, Validation, Methodology, Formal analysis, Data curation. **Tatsuro Iso:** Investigation, Formal analysis. **Huaichun Wen:** Investigation. **Anqi Zhang:** Investigation. **Tetsuya Hatakeyama:** Investigation. **Hiraku Oshima:** Software, Data curation. **Tsunehiro Mizushima:** Writing – review & editing, Writing – original draft, Visualization, Validation, Supervision, Methodology, Investigation, Funding acquisition, Formal analysis, Data curation, Conceptualization. **Masayuki Yamamoto:** Writing – review & editing, Writing – original draft, Supervision, Funding acquisition, Conceptualization.

## Declaration of competing interest

None declared

## Data Availability

Data will be made available on request.
