# Supplementary material for: Bidirectional regulation of KEAP1 BTB domain-based sensor activity
Source: Redox Biol. 2025 Oct 8;87:103885. doi: 10.1016/j.redox.2025.103885 (PMC12550724; doi:10.1016/j.redox.2025.103885)
Supplement: Multimedia component 1 [file mmc1.pdf]

Supplementary Table S1

	KEAP1 BTB WT	KEAP1 BTB CDDO-Im	KEAP1 BTB CDDO-Me
**Data Collection**			
Resolution (Å)	37.08 - 2.80 (2.95 - 2.80)	42.52 - 2.44 (2.59 - 2.44)	46.26 - 3.43 (3.57 - 3.43)
Space group	$P\,6_5\,2\,2$	$P\,2_1$	$C\,2$
Cell dimensions			
a, *b*, *c* (Å)	42.8 42.8 261.9	73.6 79.4 110.9	324.5 72.1 92.7
α, β, γ (°)	90 90 120	90 94.6 90	90 103.3 90
Number of reflections measured	51081 (7613)	163012 (27036)	94123 (10191)
Number of unique reflections	6661 (942)	88627 (14786)	50654 (5563)
Redundancy	7.7 (8.1)	1.8 (1.8)	1.9 (1.8)
Completeness (%)	99.9 (99.8)	98.0 (99.0)	97.03 (94.72)
$\langle I/\sigma \rangle$	14.32 (1.79)	11.07 (1.93)	10.83 (1.61)
R_{merge}	0.07812 (1.269)	0.03181 (0.2695)	0.0294 (0.3544)
$CC_{1/2}$	0.999 (0.750)	0.998 (0.884)	0.999 (0.943)
**Refinement**			
Number of reflections	4067 (543)	46708 (7815)	27485 (2960)
R_{work} (%)	0.2225 (0.2666)	0.2118 (0.2753)	0.2535 (0.5240)
R_{free} (%)	0.2608 (0.3992)	0.2501 (0.3142)	0.3200 (0.5738)
**No. of atoms**			
Protein	1018	8078	7627
Ligand/ion	0	282	185
Water	0	0	0
**RMSDs**			
Bond lengths (Å)	0.010	0.010	0.008
Bond angles (°)	1.12	1.70	0.98
**Ramachandran Plot Statistics (%)**			
Favored regions	93.75	97.74	90.84
Allowed regions	5.47	1.97	8.11
Disallowed regions	0.78	0.29	1.05
**Average B-factors (Å)**			
Overall	96.09	76.50	195.55
Protein	96.09	76.52	195.31
Ligand/ion	-	75.93	205.43
Water	-	-	-

Each dataset was collected from a single crystal. Values in parenthese are for the highest resolution shell. RMSD, root-mean-square deviation. Statistics for this table were generated by program Phenix.
