# Supplementary material for: Bidirectional regulation of KEAP1 BTB domain-based sensor activity
Source: Redox Biol. 2025 Oct 8;87:103885. doi: 10.1016/j.redox.2025.103885 (PMC12550724; doi:10.1016/j.redox.2025.103885)

Supplementary figure legends

Supplementary Figure S1.

Purification of the KEAP1 BTB domain to homogeneity by cloning the KEAP1-BTB domain (50-179 amino acids with S172A substitution) into the pET101 vector and expression in E. coli. KEAP1-BTB domain was subsequently purified. 6xHis-tagged BTB protein was purified using HisPur Ni-NTA Resin. (**A**) The purified 6xHis-tagged BTB was digested with TEV protease, and the untagged BTB protein was obtained by removing the 6xHis-tag and purified by gel filtration. All of the proteins were near homogeneity, as judged by sodium dodecyl sulfate-polyacrylamide gel electrophoresis (SDS-PAGE) stained with Oriole Fluorescent Gel Stain. (**B**) Purification of BTB protein homodimer to homogeneity verified by gel filtration.

Supplementary Figure S2.

Electron density maps ($2F_o$-F_c (sigma level 1.0: black), F_o-F_c (sigma level +/-3.0: green/red) and Polder map (sigma level 3.0: black)) showing CDDO-Im (yellow) and the surrounding residues Cys151 and Tyr85.

Supplementary Figure S3.

Reaction model showing that CDDO-Im binds to both Cys151 and Tyr85 of KEAP1 removing an imidazole moiety of CDDO-Im.

Supplementary Figure S4.

Electron density maps ($2F_o$-F_c (sigma level 1.0: black), F_o-F_c (sigma level +/-3.0: green/red) and Polder map (sigma level 3.0): black) showing CDDO-Me (salmon) and the surrounding residues Cys151 and Tyr85 (magenda).

Supplementary Figure S5.

(**A**) Co-crystal structure (PDB 5NLB) of the KEAP1-BTB and CUL3. Note that Part 1 (loop 112-121) interacts with CUL3. (**B**) Superimposed structures of Part 1 (loop 112-121) of the

KEAP1-BTB domain with CDDO-Im (PDB 9UJI). Note that the Part 1 loop appears highly variable among eight molecules in asymmetric unit with CDDO-Im. (**C**) Co-crystal structure (PDB 5NLB) of the KEAP1-BTB and CUL3. Note that Part 1 (loop 112-121) interacts with CUL3. (**D**) Superimposed structures of Part 1 (loop 112-121) of the KEAP1-BTB domain with CDDO-Im (PDB 9UJI). Note that the Part 1 loop appears highly variable among eight molecules in asymmetric unit with CDDO-Im.

Supplementary Figure S6.

Phylogenetic alignment of the KEAP1-BTB domains. Note that Tyr85, H129, Cys151 and H154 are highly conserved among vertebrates. Multiple sequence alignment was performed using Clustal Omega [59]. The secondary-structure assignment and figure were produced using ESPript [60].

References

58. Sievers, F. et al. Fast, scalable generation of high-quality protein multiple sequence alignments using Clustal Omega. *Mol Syst Biol* **7**, 539 (2011).

59. Robert, X. & Gouet, P. Deciphering key features in protein structures with the new ENDscript server. *Nucleic Acids Res* **42**, W320-4 (2014).

Supplementary Figure S1

A

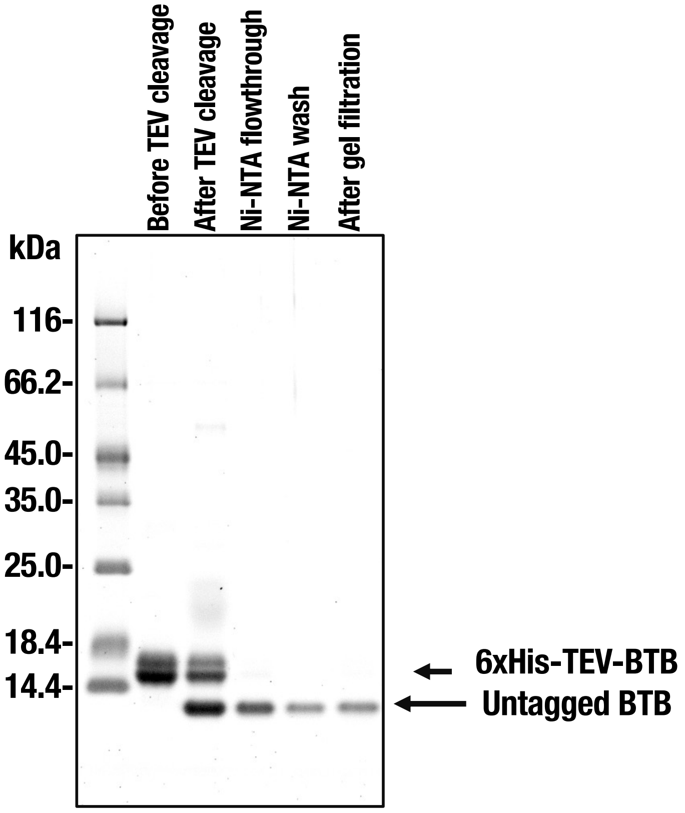

B

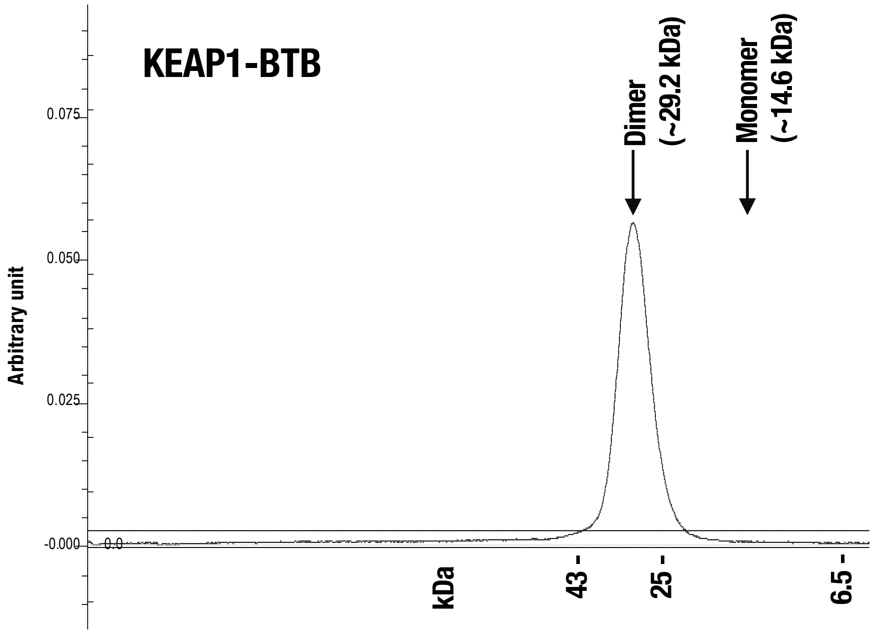

Supplementary Figure S2

$2F_o$-F_c (sigma level : 1.0)
F_o-F_c (sigma level : +/- 3.0)

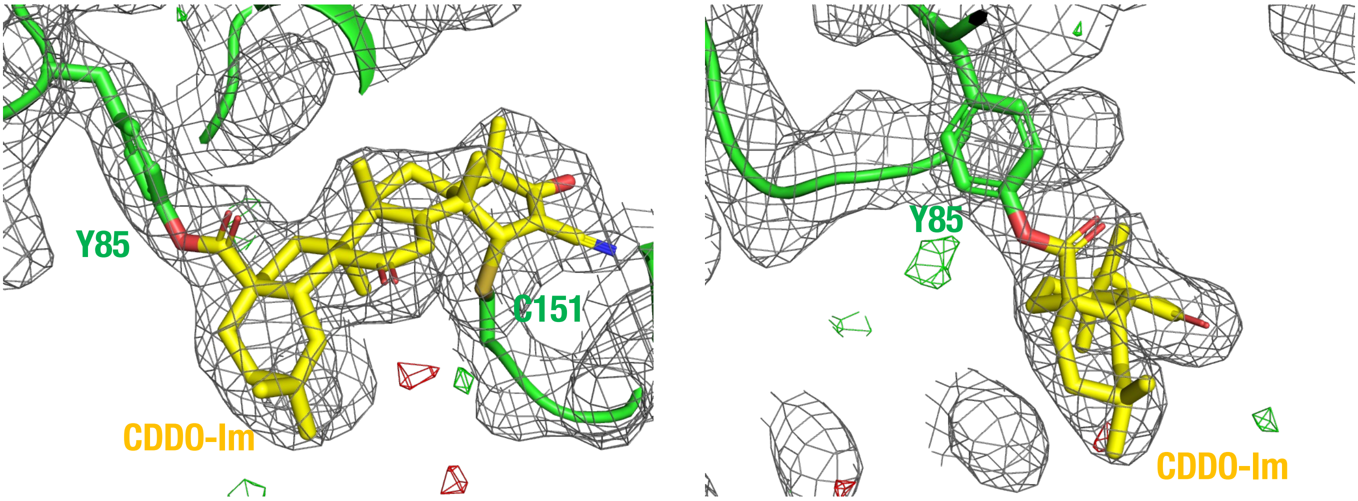

Polder map (sigma level : 3.0)

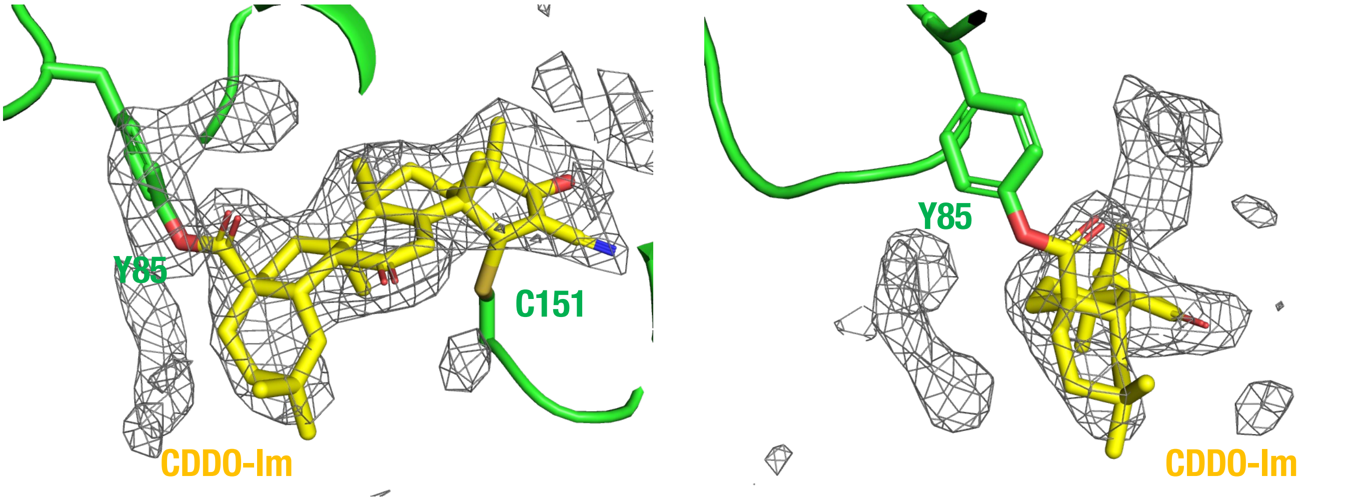

Supplementary Figure S3

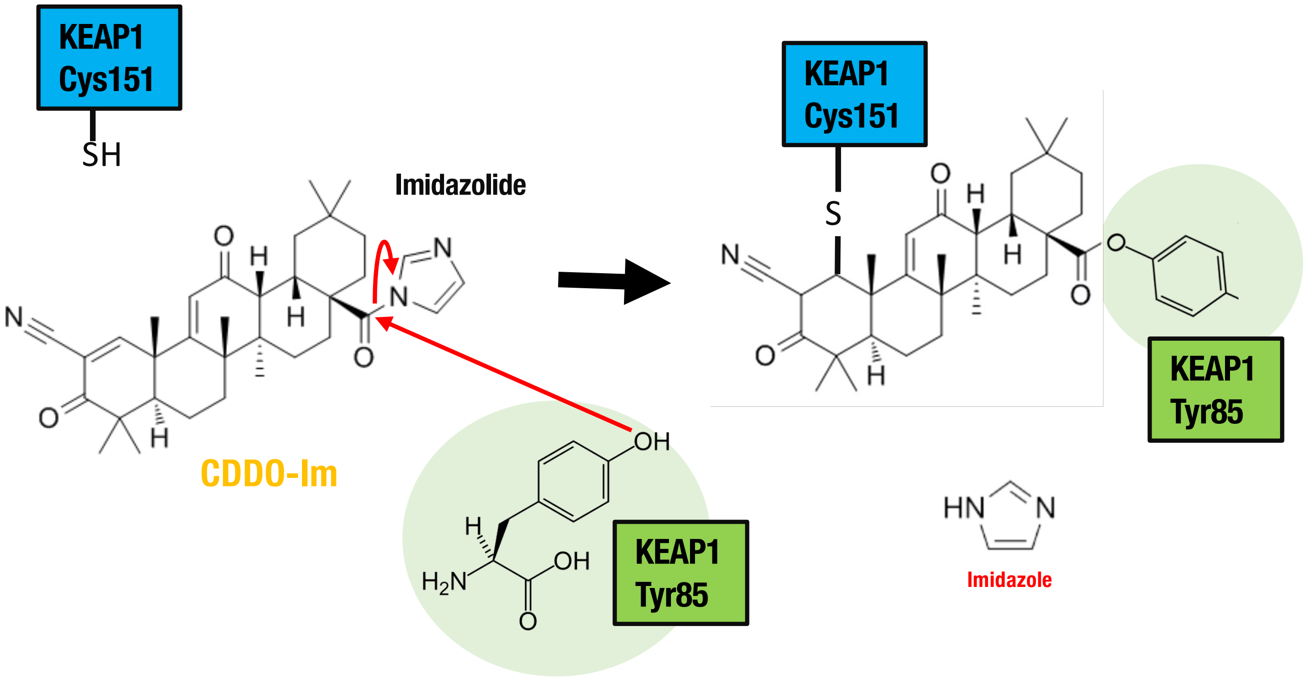

Supplementary Figure S4

$2F_o$-F_c (sigma level : 1.0)

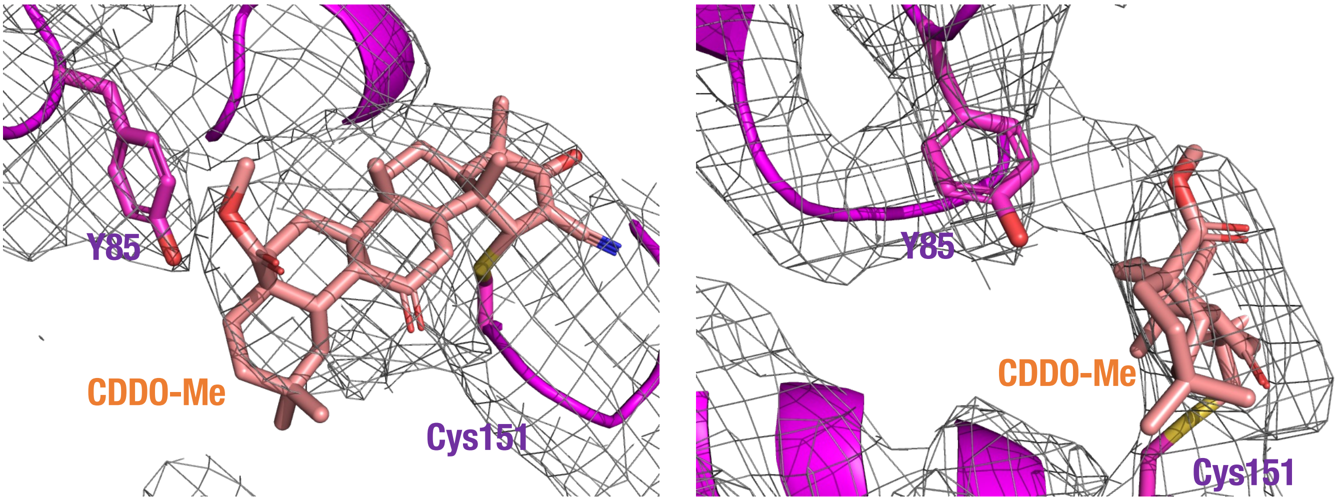

$2F_o$-F_c (sigma level : 1.0)
F_o-F_c (sigma level : +/- 3.0)

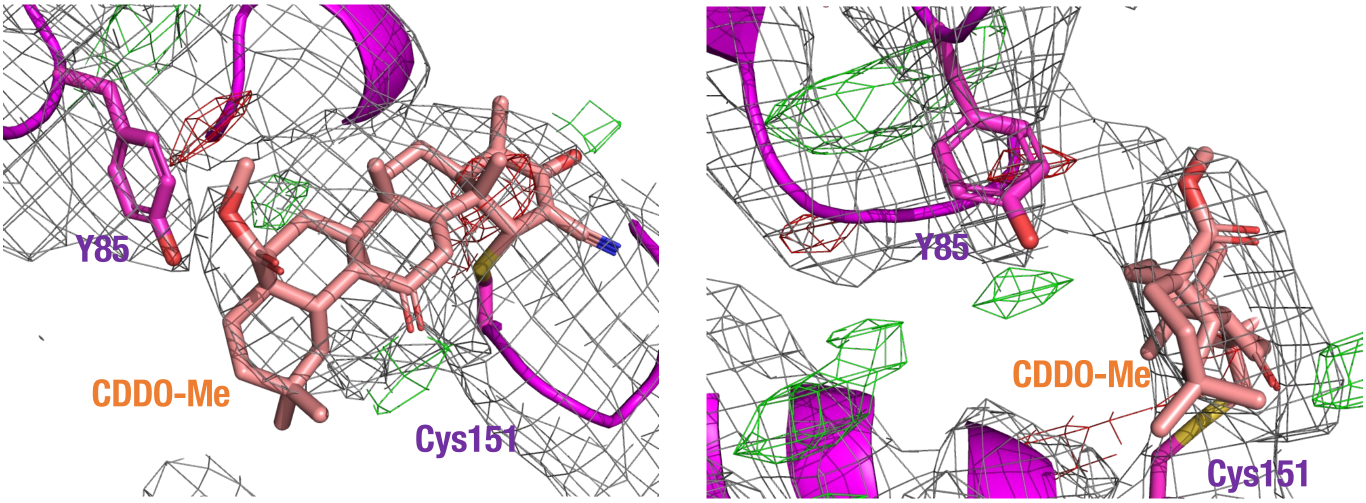

Polder map (sigma level : 3.0)

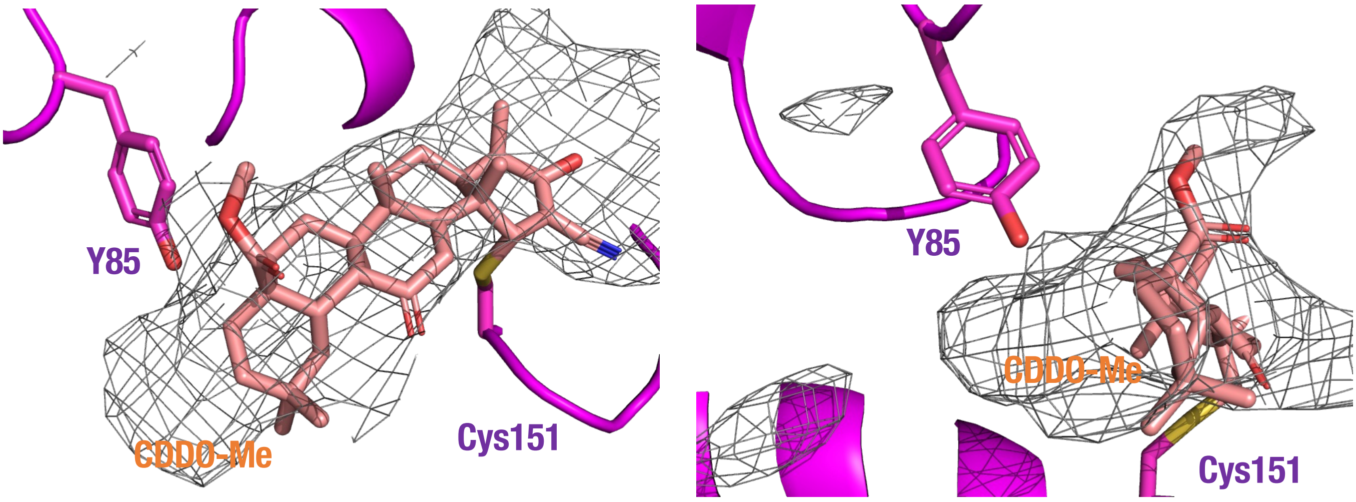

Supplementary Figure S5

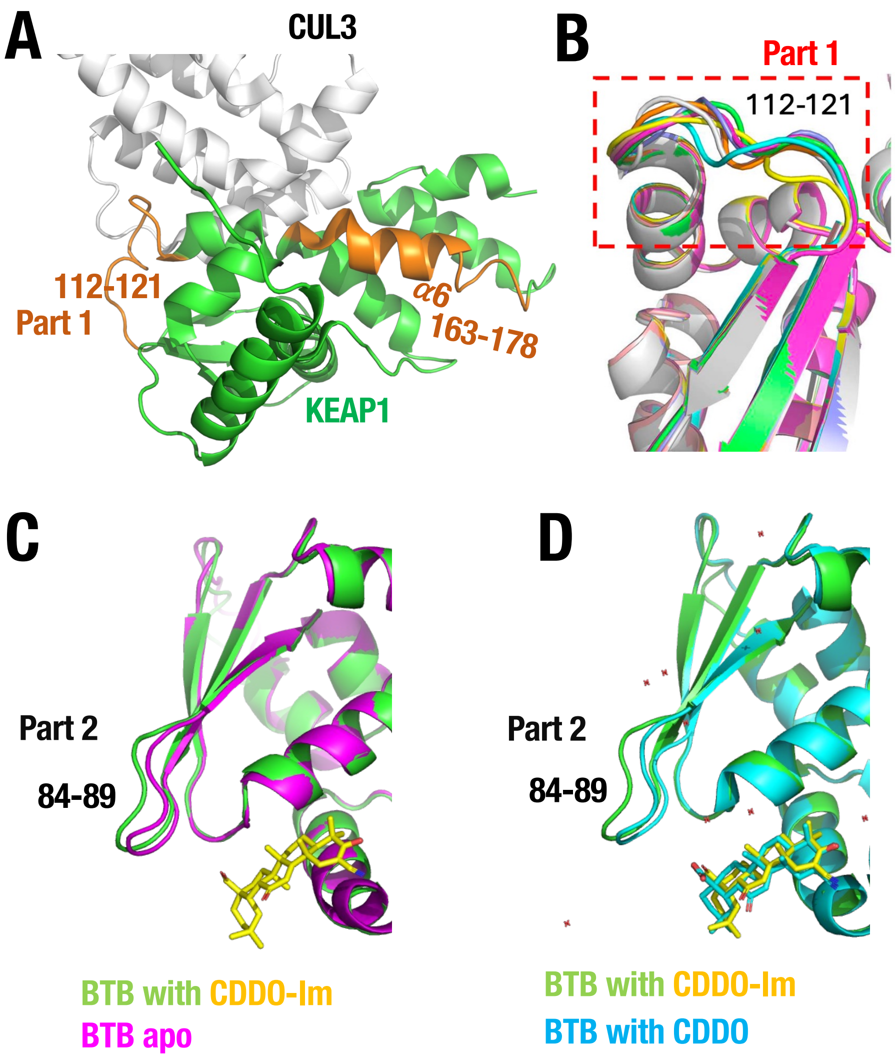

Supplementary Figure S6

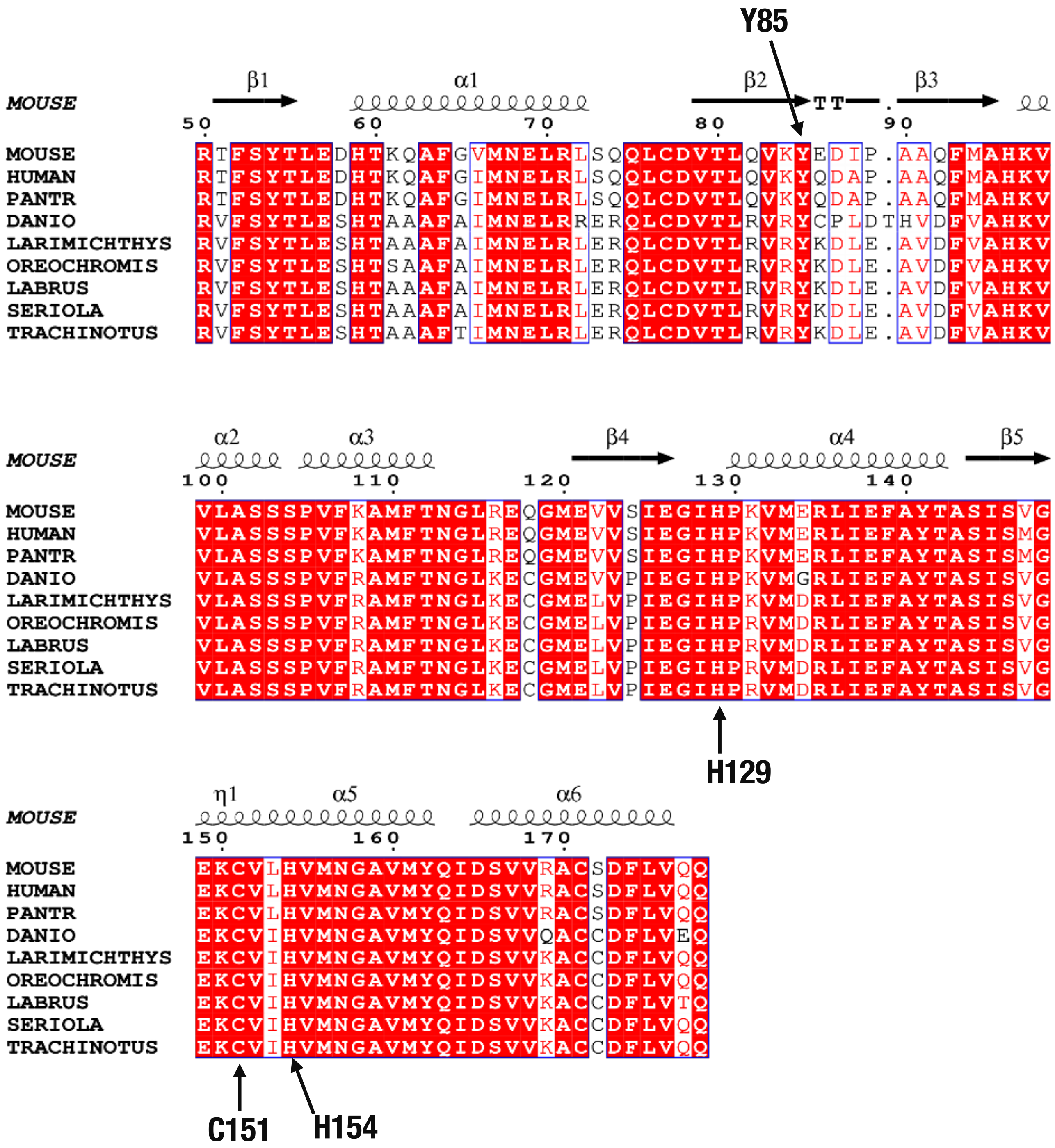

Supplement: Multimedia component 2 [file mmc2.pdf]
